# Research progress and hotspots of the impact of Mediterranean diet on brain health from 2005 to 2025: a bibliometric and visualization analysis

**DOI:** 10.3389/fnut.2026.1796774

**Published:** 2026-03-17

**Authors:** Bingya Zhang, Shuai Hu, Hui Li

**Affiliations:** 1 Kunshan Integrated TCM and Western Medicine Hospital, Suzhou, China; 2 Physical Education and Sports School of Soochow University, Suzhou, China

**Keywords:** Alzheimer’s disease, analysis, bibliometric, brain, cognitive impairment, depression, diet, health

## Abstract

**Objective:**

To conduct bibliometric and visual analysis in the field of Mediterranean diet (MD) on brain health from 2005 to 2025.

**Methods:**

Relevant literature published between 2005 and 2025 was retrieved from the Web of Science and Scopus databases. R software and VOSviewer were used for data analysis and visualization.

**Results:**

The number of publications in this field showed a steady annual increase. The United States ranked first in terms of publication output, followed by Italy, Spain, China, and Australia. Notably, the United States also played a prominent role in international collaboration, with institutions such as Harvard University and the University of Barcelona actively cooperating with research centers worldwide. At the journal level, *Nutrients* emerged as the core academic platform in this field, ranking first in both the number of publications and citations. The study identified key researchers including Scarmeas N and Aggarwal NT. Major keywords included Alzheimer disease, dementia, gut microbiota, cognitive impairment, and oxidative stress, which reflect the central themes and research trends in MD and brain health.

**Conclusion:**

As a healthy dietary therapy, the MD holds great promise for significant advances in the field of brain health. Growing global attention to this dietary pattern highlights its potential to become a core research direction in dietary interventions for brain disorders. This study provides a comprehensive analysis of the current research landscape and key hotspots of MD on brain health, offering valuable references for future investigations.

## 1 Introduction

The brain is a vital organ in the human body responsible for core functions such as cognition, emotional regulation, body temperature maintenance, neural signal transmission, and the regulation of various physiological activities. The health of the brain is directly related to the maintenance of normal physiological processes, and brain diseases have therefore become a major health concern that cannot be ignored (1). The consumption of high-sugar and high-calorie diets in modern lifestyles, combined with insufficient physical activity, has jointly driven the continuous increase in the incidence of neurodegenerative diseases (2), cerebrovascular diseases (3), and depression (4). These brain disorders not only severely disrupt patients’ daily lives and social functions but also pose significant health risks and reduce life expectancy. Cognitive impairment, one of the most common clinical manifestations of brain diseases, is closely associated with Alzheimer’s disease (AD), Parkinson’s disease (PD), and depression (5). Although these conditions may not present obvious cognitive decline or emotional disturbances in their early stages, failure to detect them promptly and implement standardized interventions may lead to further progression, resulting in more severe pathological damage to the brain, including significant brain atrophy and the development of dementia (6, 7).

Lifestyle interventions play a crucial role in maintaining brain health and preventing or ameliorating neurodegenerative diseases (8). In terms of dietary adjustments, increasing the intake of whole grains, fruits, and vegetables offers significant benefits for brain health (9). Regular aerobic exercise exerts neuroprotective effects and effectively improves cognitive function (10). Pharmacological treatment is also an important approach to managing cognitive impairment, including cholinesterase inhibitors, NMDA receptor antagonists, selective serotonin reuptake inhibitors, vitamin E, and glutamate receptor antagonists (11, 12). However, the effectiveness of lifestyle interventions is often limited by patient compliance, and improvements in brain health may take a long time to become evident. Meanwhile, pharmacological interventions may be accompanied by potential adverse effects (13). For AD, there remains a lack of specific therapeutic drugs. In recent years, breakthroughs have been made in immunotherapy, which aims to activate the immune system to clear accumulated *β*-amyloid (Aβ) proteins in the brain, thereby improving cognitive function (14). In addition, non-invasive brain stimulation techniques such as repetitive transcranial magnetic stimulation (rTMS) can be used as adjunctive treatments to alleviate dementia symptoms, although not all patients achieve satisfactory outcomes (15).

Treatment in the early stages of PD is mainly based on levodopa, with the core goal of delaying the occurrence of motor complications. Clinically, levodopa/carbidopa intestinal gel (LCIG) achieves individualized and stable blood drug concentrations through continuous delivery and is primarily used to treat motor complications in patients with mid-to-late-stage PD (16). UCB0599, an oral drug, can selectively inhibit the aggregation of *α*-synuclein (17). However, the progression of PD remains largely irreversible, and current treatments mainly focus on controlling and alleviating symptoms. Patients with depression have a significantly increased risk of suicide. Those with major depressive disorder often experience intense suicidal ideation, which is one of the leading causes of death related to brain diseases, and their treatment remains highly challenging (18). Antidepressant medications and psychotherapy are first-line interventions. Drugs such as escitalopram and duloxetine have been shown to effectively improve depressive symptoms (19). However, factors such as differences in the severity of depression, individual variability, and adverse effects may influence treatment outcomes. Psychotherapy also faces the challenge of limited efficacy in patients with moderate to severe depression (20). Therefore, exploring innovative intervention strategies is crucial for improving the treatment of brain diseases.

The Mediterranean diet (MD) is centered on plant-based foods such as fruits, vegetables, fish, whole grains, legumes, and olive oil, emphasizing dietary diversity and nutritional balance. This dietary pattern is rich in dietary fiber, monounsaturated fats, vitamins, and minerals, which can meet the comprehensive nutritional needs of the human body. In recent years, the MD has attracted considerable attention due to its potential health benefits, particularly in promoting brain health (21). By reducing the intake of refined carbohydrates, it helps lower blood glucose levels and improve insulin sensitivity (22), which in turn has a positive impact on brain energy metabolism and may assist in the management of neurodegenerative diseases (23). Furthermore, the MD can reduce blood pressure and protect cerebrovascular function by mitigating inflammatory responses in the vascular wall, thereby decreasing diffuse cerebral microinfarcts and improving cerebral perfusion (24). Improved cerebral blood flow also helps reduce neuronal apoptosis and prevent progression to dementia (25). At the same time, the MD’s regulatory effects on neurotransmitter synthesis and metabolism make it a promising intervention for depression (26). This dietary pattern provides abundant B vitamins, folate, tryptophan, and other nutrients that are essential for the synthesis of neurotransmitters such as serotonin and dopamine, which are closely related to emotional regulation (27). In addition, the MD helps regulate the abundance and diversity of gut microbiota, potentially exerting neuroprotective effects through the “gut microbiota–gut–brain axis” (28, 29).

Nowadays, despite the existence of numerous studies on the MD in the field of brain health, the current research has obvious limitations. Specifically, most studies focus on the interventional effects of MD on a single disease (e.g., AD or depression) and the underlying mechanisms involved (e.g., oxidative stress, the gut-brain axis), while a comprehensive and integrated analysis of the developmental trends, research priorities and hotspots of MD in brain health is still lacking. Bibliometric analysis enables the quantitative evaluation of scientific publications and the identification of specific research trends, thereby facilitating a better understanding of the current research progress and future directions (30). Therefore, to more accurately characterize the research progress and developmental trends of MD in the field of brain health, based on the availability and comprehensive coverage of data and drawing on existing studies, we conducted a visual analysis of relevant literature published over the past two decades (2005–2025) starting from 2005, by applying visualization tools including VOSviewer, R and CiteSpace. This paper summarizes and discusses the research hotspots and trends of MD in the field of brain health, aiming to provide valuable references for researchers in this field in the future.

## 2 Materials and methods

### 2.1 Literature sources and search strategy

The Web of Science Core Collection (WoSCC) and Scopus were selected as the data sources based on their comprehensive coverage of peer-reviewed literature, high-quality content, and widespread application in bibliometric studies. WoSCC provides robust citation data and is particularly strong in clinical and biomedical research, whereas Scopus offers broader interdisciplinary coverage, thereby ensuring a more thorough retrieval of studies relevant to the Mediterranean diet and brain health. By analyzing the databases separately, we aimed to preserve data integrity and enhance the reliability of the findings while minimizing potential data loss due to differences in data formatting and indexing standards. On January 4, 2026, literature searches were conducted in the WoSCC and Scopus databases. In WoSCC, the search strategy was defined as follows: TS = (Mediterranean diet* OR Mediterranean dietary pattern) AND TS = (Brain OR Encephalon* OR Cerebrum* OR Central nervous system* OR cranial nerve*) AND PY = (2005–2025) AND DT = (Article OR Review) AND LA = (English). In Scopus, the search strategy was defined as: TITLE-ABS-KEY (“Mediterranean diet” OR “Mediterranean dietary pattern”) AND TITLE-ABS-KEY (“Brain” OR “Encephalon” OR “Central nervous system”) AND PUBYEAR > 2004 AND PUBYEAR < 2026 AND (LIMIT-TO (DOCTYPE, “ar”) OR LIMIT-TO (DOCTYPE, “re”)) AND (LIMIT-TO (LANGUAGE, “English”)). Records retrieved from WoSCC were saved in plain text format and exported as full records, including cited references. Records retrieved from Scopus were saved in CSV format and exported as full records, also including cited references.

### 2.2 Inclusion and exclusion criteria

**Figure 1:**
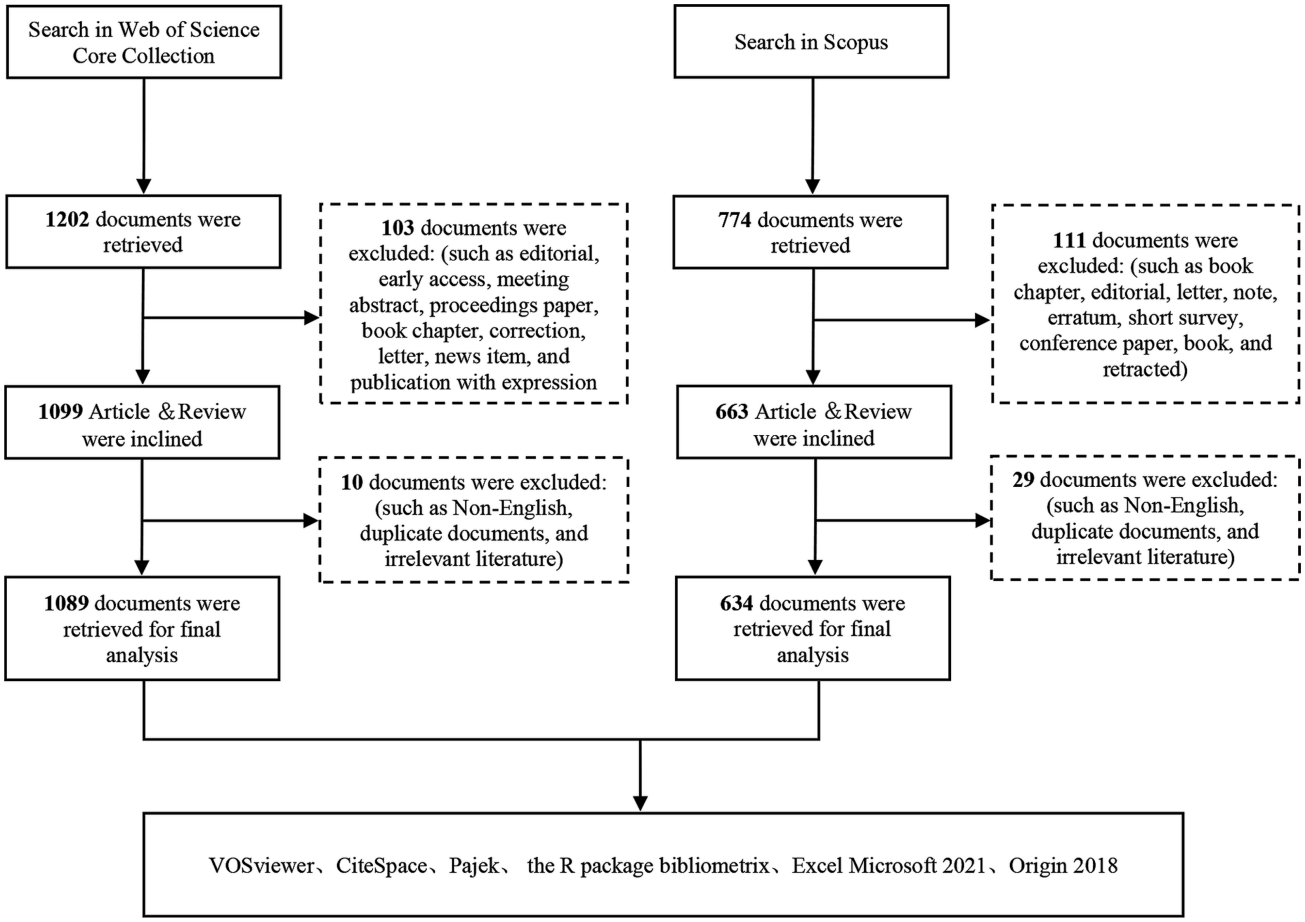
Flowchart of the publications selection.

Inclusion criteria: All English original articles and reviews related to “Mediterranean diet” and “brain.” Exclusion criteria: Duplicated literature, literature irrelevant to studies on “Mediterranean diet” and “brain,” meeting abstracts, proceeding papers, early access articles, editorial materials, book chapters, retracted publications, and letters. Notably, survey studies and cohort studies were retained as core subtypes of “Article” in the present study, as they represent the main epidemiological research designs for exploring the association between the MD and brain health. Finally, a total of 1,089 papers were included from the WoSCC database and 634 papers from the Scopus database. Figure 1 shows the flowchart of the literature search and screening process.

### 2.3 Data analysis

Given the differences in data formats between the WoSCC and Scopus databases, merging the two datasets would result in data loss. Therefore, we analyzed the data from each database separately to obtain more reliable results. In addition, it should be noted that, considering the high-quality nature of the literature indexed in WoSCC, the subsequent analyses focused primarily on the WoSCC data. The analytical results from the Scopus database, including annual publication trends and keyword clustering, are provided in the Supplementary materials. For the analysis of annual publication output, Origin 2018 software was used. Furthermore, R software (version 4.3.1) with the bibliometrix package (31), VOSviewer (version 1.6.18), and CiteSpace (version 6.4. R1) were employed to perform visual analysis and construct scientific knowledge maps.

VOSviewer was used to generate visualizations of country collaboration networks, source co-citation analysis, and keyword co-occurrence. The parameter settings were as follows: minimum number of documents per country ≥ 11; minimum number of documents per institution ≥ 10; minimum number of documents per author ≥ 15. For source co-citation analysis, the minimum number of citations per source was set to ≥ 50. In keyword co-occurrence analysis, the parameters were set as follows: minimum number of occurrences of a keyword ≥ 10. To ensure the accuracy and interpretability of the network, we performed two preprocessing steps: First, general search terms—namely “Mediterranean diet,” “brain,” and their variants—were excluded. Since these terms appear in nearly all retrieved documents due to the search strategy, their inclusion would artificially dominate the co-occurrence network and mask the associations between specific thematic keywords. Second, synonymous keywords were identified and merged into unified representative terms. For example, “Alzheimer’s disease,” “Alzheimer,” and “AD” were standardized as “Alzheimer disease”; “gut microbiome” and “intestinal microbiota” were unified as “gut microbiota.” This manual curation step avoided the fragmentation of related conceptual terms and ensured that the visual clustering could accurately reflect the core research themes. CiteSpace was used to identify the top 25 cited references with the strongest citation bursts. The CiteSpace parameters were set as follows: time slice (2005–2025), number of years per slice (1), node type (cited references), selection criteria (top *N* = 50), and no pruning. Journal impact factor (IF) data were obtained from the 2024 Journal Citation Reports (JCR).

## 3 Results

### 3.1 Overall overview of the included literature on the MD and brain health

**Figure 2:**
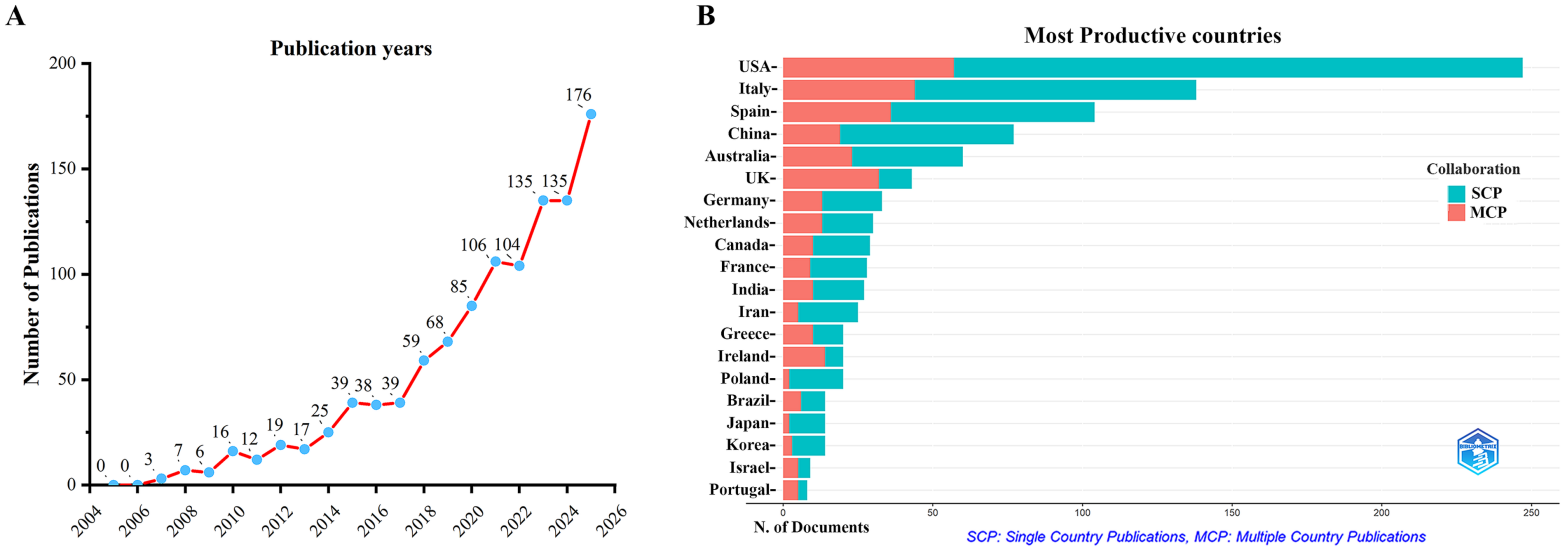
Trends in annual publication outputs on MD in brain health from 2005 to 2025. **(A)** Trends of annual publication outputs. **(B)** Distribution of corresponding authors’ countries and cooperation.

A total of 1,089 unique documents were retrieved from the Web of Science Core Collection (WoSCC). As shown in Figure 2A, the annual number of publications in this field generally exhibited an upward trend from 2005 to 2025. Notably, the growth rate was particularly significant between 2022 and 2025. The year 2025 saw the highest number of publications (176 articles), further enriching the research output in this domain. A total of 634 unique records were collected from the Scopus database (after removing duplicates), and the trend in publication output was consistent with that of WoSCC (Supplementary Figure S1). These findings indicate a growing interest in the relationship between the MD and brain health in recent years.

**Figure 3:**
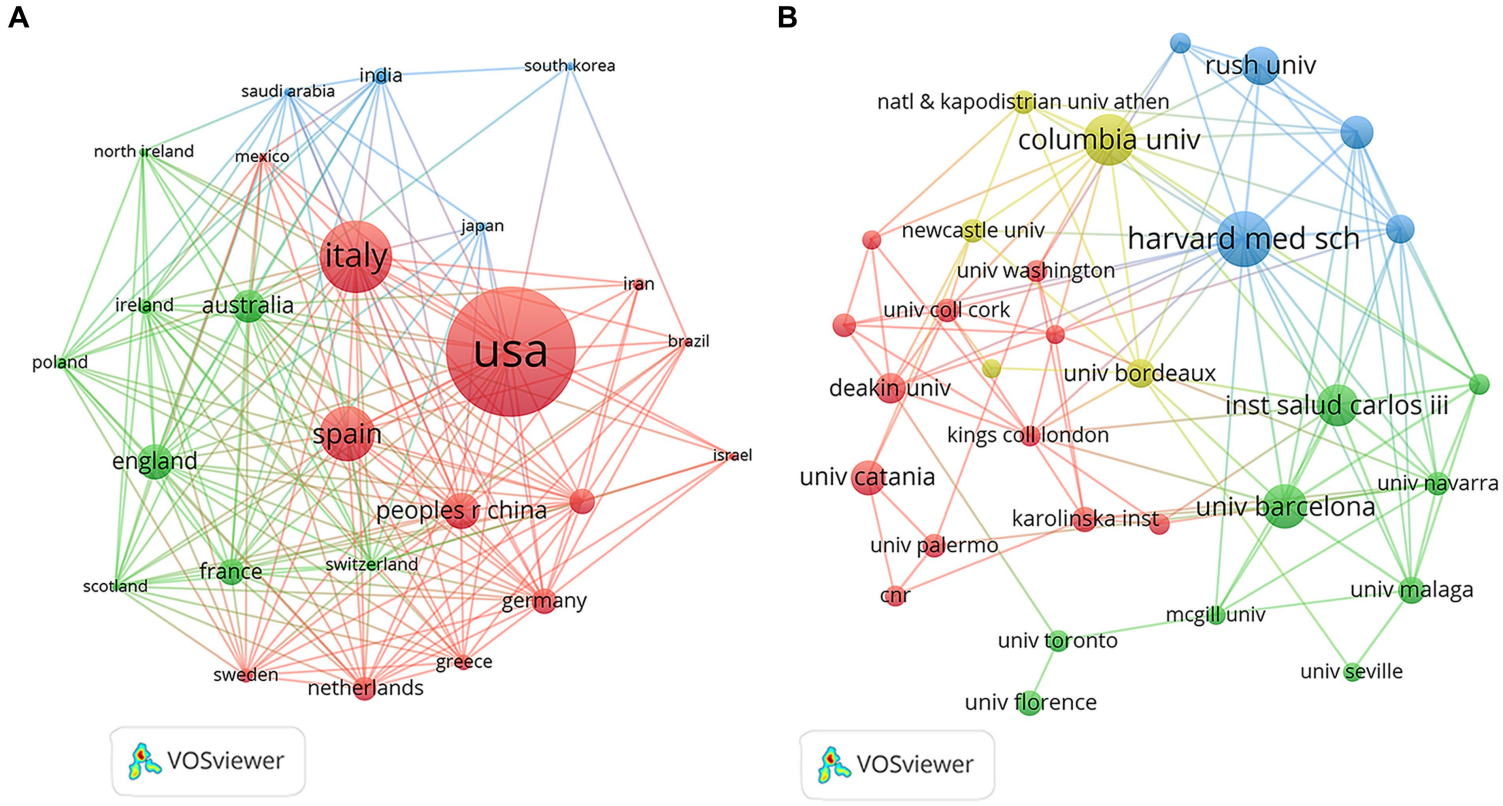
Map of countries/regions and institutions involved in MD in brain health from 2005 to 2025. **(A)** Map of international cooperation networks. **(B)** Map of institutional cooperation networks.

The top five countries in terms of publication output in this field were the United States (*n* = 247), Italy (*n* = 138), Spain (*n* = 104), China (*n* = 77), and Australia (*n* = 50). Among them, the United States published the largest number of papers, indicating its leading position and significant contributions to the field. In addition, among the top 10 countries in terms of publication volume, the United Kingdom (74.4%) and the Netherlands (43.3%) had the highest proportions of multi-country collaborative papers (MCPs), suggesting that these two countries attach great importance to international cooperation and academic exchange. In contrast, the United States (23.1%) and China (24.7%) had relatively low proportions of multi-country collaborative papers, indicating that their research efforts place greater emphasis on domestic original work (Figure 2B; Table 1). Furthermore, Figure 3A shows that the United States is the central hub of collaboration in this field, highlighting its important role in driving the development of research in this area. Among the top 10 research institutions in terms of publication output, five were from the United States, two from Spain, and one each from Italy, Australia, and France. Harvard University in the United States published the largest number of papers (*n* = 35), followed by Columbia University in the United States (*n* = 32) and the University of Barcelona in Spain (*n* = 28; Figure 3B; Table 2).

**Table 1:** Most relevant countries by corresponding authors of MD on brain health.

Country	Articles	SCP	MCP	Freq (%)	MCP_Ratio (%)
USA	247	190	57	22.7	23.1
Italy	138	94	44	12.7	31.9
Spain	104	68	36	9.6	34.6
China	77	58	19	7.1	24.7
Australia	60	37	23	5.5	38.3
United Kingdom	43	11	32	3.9	74.4
Germany	33	20	13	3	39.4
Netherlands	30	17	13	2.8	43.3
Canada	29	19	10	2.7	34.5
France	28	19	9	2.6	32.1

MCP, Multiple country publication; SCP, Single country publication.

**Table 2:** Top 10 most relevant affiliations of MD on brain health.

Rank	Affiliation	Articles (n)
1	Harvard University	141
2	University of Barcelona	105
3	Rush University	93
4	University of California System	77
5	Columbia University	73
6	Harvard University Medical Affiliates	65
7	Ciber - Centro De Investigacion Biomedica En Red	58
8	Hospital Clinic De Barcelona	57
9	University of Catania	54
10	University of Illinois System	53

### 3.2 Journals and co-cited journals

**Figure 4:**
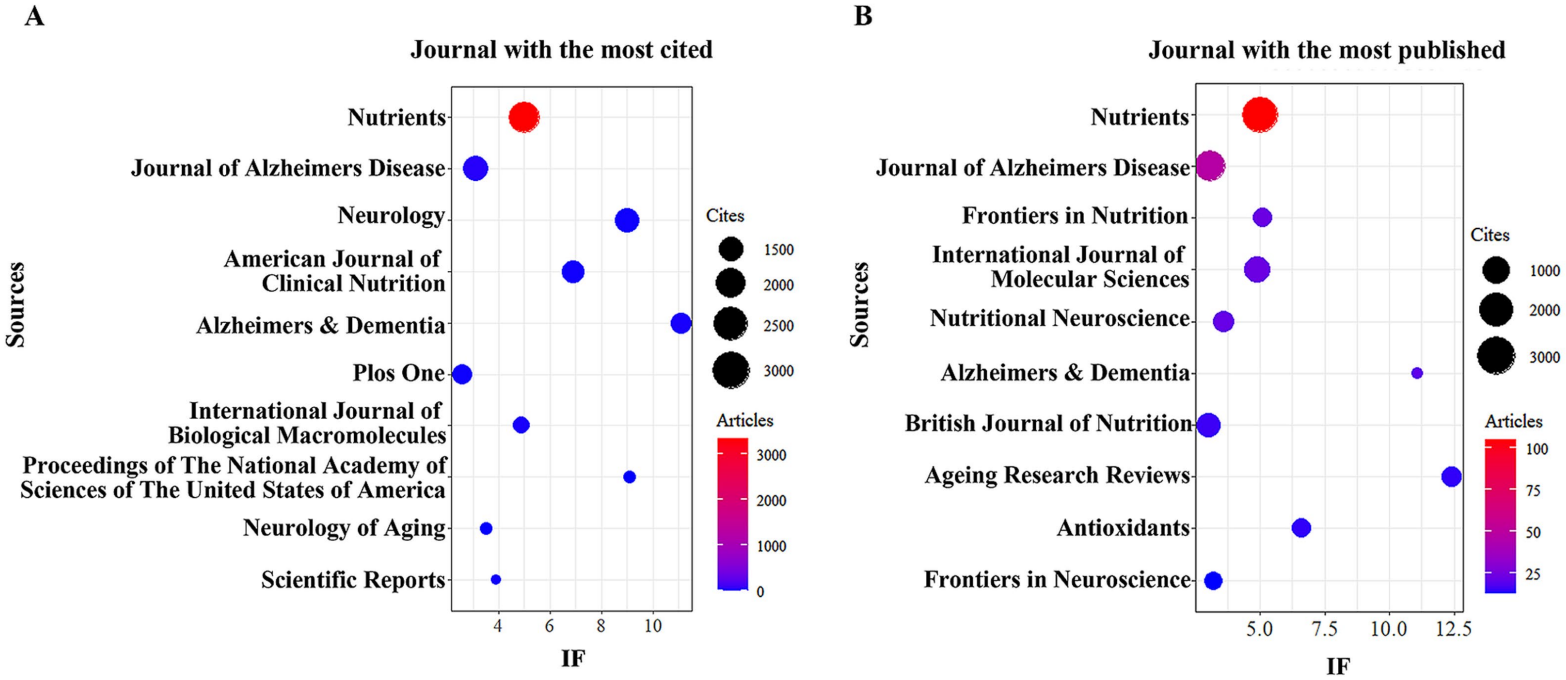
Journals with the highest publication volume and citation counts. **(A)** Journals with the highest citation counts. **(B)** Journals with the highest publication volume.

The bibliometrix and ggplot2 packages in R software were used to analyze the most cited articles and journals in this field. In addition, VOSviewer was employed for co-citation journal analysis. The results showed that the 1,089 papers were distributed across 415 academic journals (Table 3). Figure 4A shows that the journal with the highest number of citations was *Nutrients* (*n* = 3,347, IF = 5), followed by *Journal of Alzheimer’s Disease* (*n* = 2,308, IF = 3.1), *Neurology* (*n* = 2,124, IF = 9), *American Journal of Clinical Nutrition* (*n* = 1857, IF = 6.9), and *Alzheimer’s & Dementia* (*n* = 1,628, IF = 11.1).

**Table 3:** Top 10 journals with the most cited journals.

Rank	Journal	Cites	Articles	IF (2024)	JCR partition (2024)
1	Nutrients	3,347	3,347	5	Q1
2	Journal of Alzheimers Disease	2,308	48	3.1	Q2
3	Neurology	2,124	11	9	Q1
4	American Journal of Clinical Nutrition	1857	12	6.9	Q1
5	Alzheimers & Dementia	1,628	21	11.1	Q1
6	Plos One	1,588	10	2.6	Q1
7	International Journal of Molecular Sciences	1,237	23	4.9	Q1
8	Proceedings of The National Academy of Sciences of The United States of America	1,055	0	9.1	Q1
9	Neurobiology of Aging	1,046	3	3.5	Q2
10	Scientific Reports	1,012	12	3.9	Q1

**Figure 5:**
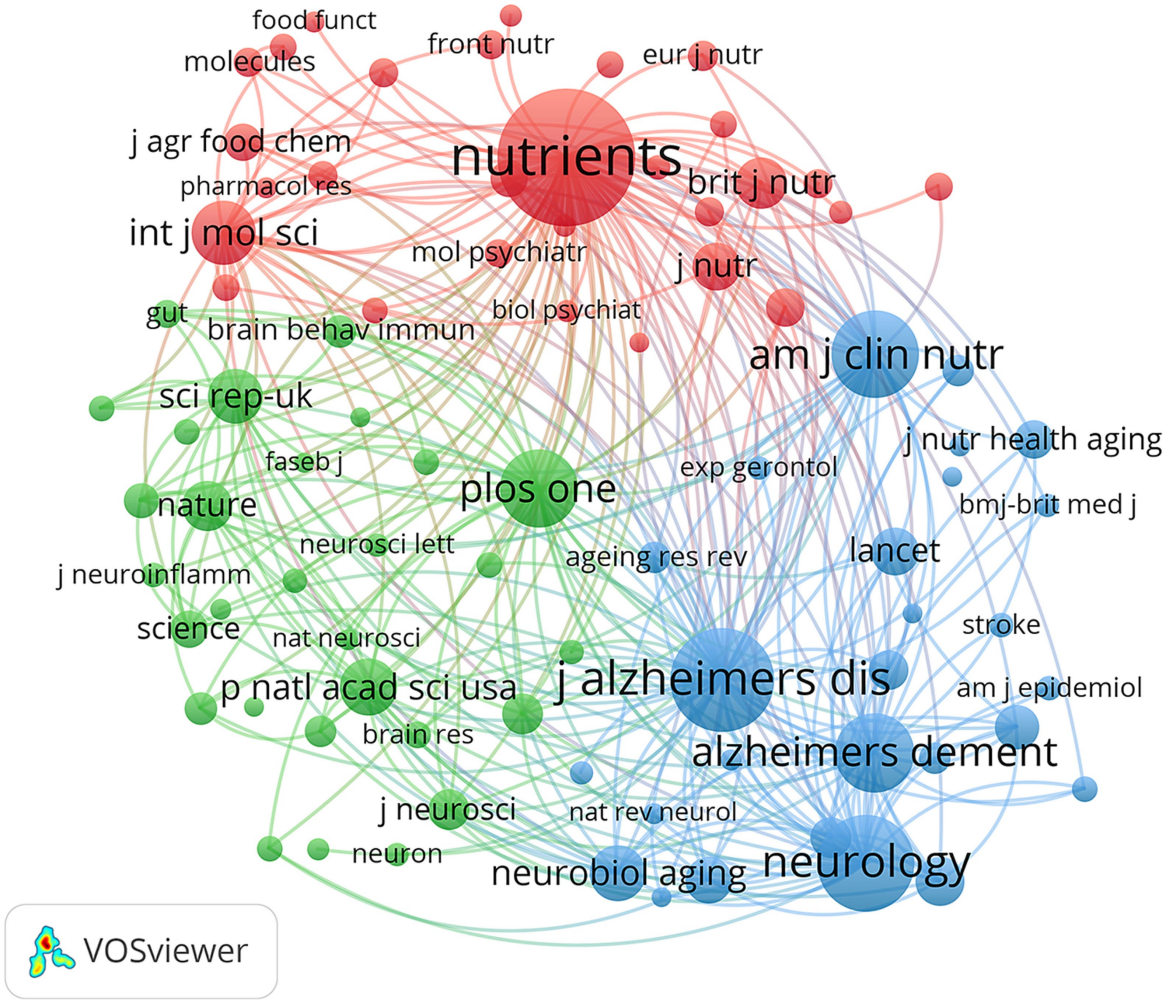
Co-cited journals related to MD in brain health from 2005 to 2025.

Table 4 and Figure 4B show that the journal with the largest number of publications was *Nutrients* (*n* = 105, IF = 5), followed by *Journal of Alzheimer’s Disease* (*n* = 48, IF = 3.1), *Frontiers in Nutrition* (*n* = 23, IF = 5.1), *International Journal of Molecular Sciences* (*n* = 23, IF = 4.9), and *Nutritional Neuroscience* (*n* = 22, IF = 3.6). The co-citation journal map indicated that *Nutrients*, *Journal of Alzheimer’s Disease*, *Neurology*, *Alzheimer’s & Dementia*, and *American Journal of Clinical Nutrition* were representative collaborative hubs in this field (Figure 5). These results suggest that *Nutrients* has significant influence in the field of the MD and brain health.

**Table 4:** Top 10 journals with the most published articles.

Rank	Journal	Articles	Cites	IF (2024)	JCR partition (2024)
1	Nutrients	105	3,347	5	Q1
2	Journal of Alzheimers Disease	48	2,308	3.1	Q2
3	Frontiers in Nutrition	23	448	5.1	Q1
4	International Journal of Molecular Sciences	23	1,237	4.9	Q1
5	Nutritional Neuroscience	22	617	3.6	Q2
6	Alzheimers & Dementia	21	78	11.1	Q1
7	British Journal of Nutrition	16	923	3	Q2
8	Ageing Research Reviews	15	486	12.4	Q1
9	Antioxidants	15	400	6.6	Q1
10	Frontiers in Neuroscience	13	378	3.2	Q2

### 3.3 Author and co-author

**Figure 6:**
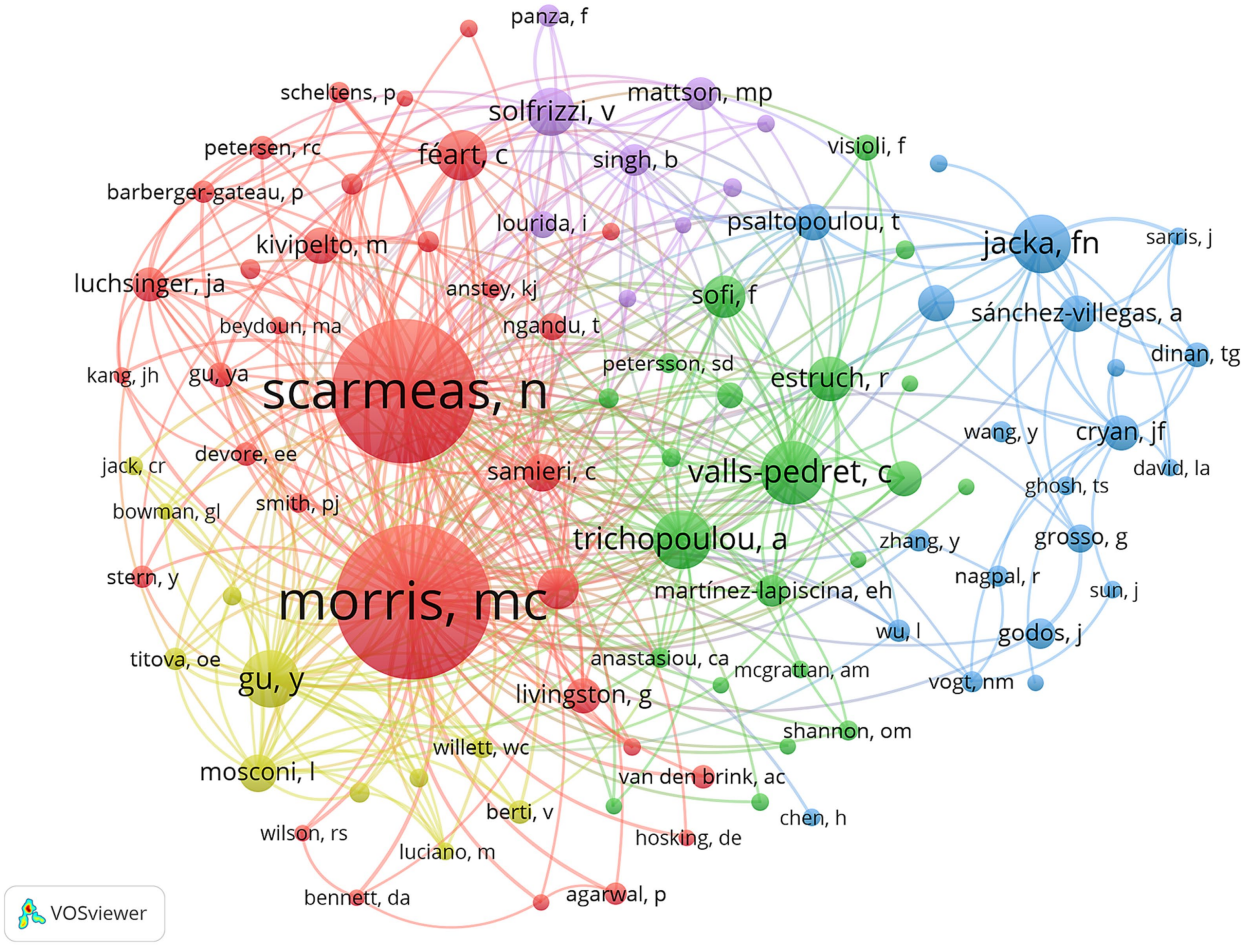
The map of co-authorship in the field of the field of MD in brain health from 2005 to 2025.

Table 5 lists the top 10 authors ranked by the number of publications. The top five authors were Scarmeas N (*n* = 18), Estruch R (*n* = 14), Grosso G (*n* = 14), Samieri C (*n* = 14), and Aggarwal NT (*n* = 13). Table 6 presents the top 10 authors with the highest number of citations. The top five authors by citation count were Scarmeas N (*n* = 375), Aggarwal NT (*n* = 260), Barnes LL (*n* = 253), Bennett DA (*n* = 227), and Morris MC (*n* = 222). The number of publications reflects an author’s research output to a certain extent, while the citation count indicates their academic influence. Notably, four researchers—Scarmeas N, Aggarwal NT, Barnes LL, and Bennett DA—appeared in both the top 10 lists for publications and citations, highlighting their important contributions and core influence in this field. In addition, most of these authors were from the United States, further emphasizing the prominent role and influence of US researchers in this domain. Furthermore, co-author network analysis (Figure 6) revealed extensive and close collaborations among authors. Scarmeas N, Morris MC, Jacka FN, Trichopoulou A, Gu Y, and Valls-pedret C emerged as representative collaborative hubs, further confirming their profound impact on the field.

**Table 5:** Top 10 documents authors related to MD on brain health.

Rank	Authors	Documents	Citations	Affiliation
1	Scarmeas N	18	375	Columbia University
2	Estruch R	14	85	Instituto de Salud Carlos III
3	Grosso G	14	42	University of Catania
4	Samieri C	14	146	University of Bordeaux
5	Aggarwal NT	13	260	Rush University
6	Ros E	13	75	University of Barcelona
7	Agarwal P	12	115	Rush University
8	Craft S	12	114	Wake Forest University School of Medicine
9	Barnes LL	11	253	Rush University
10	Bennett DA	11	227	Rush University

**Table 6:** Top 10 citations authors related to MD on brain health.

Rank	Authors	Citations	Documents	Affiliation
1	Scarmeas N	375	18	Columbia University
2	Aggarwal NT	260	13	Rush University
3	Barnes LL	253	11	Rush University
4	Bennett DA	227	11	Rush University
5	Morris MC	222	7	Rush University
6	Sacks FM	198	4	Rush University
7	Brickman AM	194	5	Columbia University
8	Stern Y	194	8	Columbia University
9	Luchsinger JA	182	4	Columbia University
10	Tangney CC	175	3	Rush University

It should be noted that core researchers in this field were mainly from research teams at Columbia University and Rush University, reflecting a certain degree of team clustering among the core research forces. Meanwhile, independent researchers including Estruch R (14 publications, 85 citations), Grosso G (14 publications, 42 citations), Samieri C (14 publications, 146 citations), Ros E (13 publications, 75 citations), and Craft S (12 publications, 114 citations) focused on relatively underexplored topics such as the MD and depression in middle-aged populations, providing unique perspectives for the field. Although independent researchers showed relatively lower citation influence due to limited resources, their work effectively filled research gaps of core teams and improved the diversity of research in this field.

### 3.4 Most cited references and reference burst

We used the bibliometrix package in R to screen out a total of 25 highly cited documents related to the MD in the field of brain health (Table 7). The analysis showed that all these documents were cited more than 247 times and published in 23 different journals, indicating that there is still room for further research in this field. Notably, among the 25 highly cited documents, *JAMA–Journal of the American Medical Association* and *Molecular Psychiatry* published the largest number of articles, with only two each. This result suggests that these two journals exert a certain influence in the field. Overall, however, no single core journal has achieved absolute dominance, and the distribution of journals remains relatively dispersed. The three most frequently cited documents were: *Diagnosis and Management of Dementia: Review*, *MIND diet slows cognitive decline with aging*, and *Physical activity, diet, and risk of Alzheimer disease*. It should be noted that the MIND diet is a hybrid dietary pattern that combines characteristics of the MD and the DASH diet (Dietary Approaches to Stop Hypertension). Therefore, although this highly cited article represents an important contribution to the field of dietary patterns and brain health, it is based on a diet extended from the MD, so its findings cannot be fully attributed to the standard MD. Further analysis revealed that all these highly cited publications were published in Q1 or Q2 journals, reflecting the high quality of the foundational literature in this field. Nevertheless, they mostly focused on macro-level reviews of the field and lacked in-depth exploration of specific research directions.

**Table 7:** Top 25 cited references related to MD on brain health.

Paper	Type	DOI	Total citations	TC per Year	JCR partition (2024)
Arvanitakis Z, 2019, Jama-J Am Med Assoc	Clinical review	10.1001/jama.2019.4782	931	116.38	Q1
Morris MC, 2015, Alzheimers Dement	Cohort study	10.1016/j.jalz.2015.04.011	726	60.50	Q1
Scarmeas N, 2009, Jama-J Am Med Assoc	Cohort study	10.1001/jama.2009.1144	664	36.89	Q1
Psaltopoulou T, 2013, Ann Neurol	Meta-analysis	10.1002/ana.23944	613	43.79	Q1
Crous-Bou M, 2017, Alzheimers Res Ther	Narrative review	10.1186/s13195-017-0297-z	504	50.40	Q1
Nagpal R, 2019, Ebiomedicine	Randomized controlled trial	10.1016/j.ebiom.2019.08.032	483	60.38	Q1
Marx W, 2021, Mol Psychiatr	Narrative review	10.1038/s41380-020-00925-x	463	77.17	Q1
Shiva S, 2007, J Exp Med	Animal study	10.1084/jem.20070198	460	23.00	Q1
Markovic AK, 2019, Molecules	Narrative review	10.3390/molecules24102001	429	53.63	Q2
Babulal GM, 2019, Alzheimers Dement	Narrative review	10.1016/j.jalz.2018.09.009	411	51.38	Q1
Sandhu KV, 2017, Transl Res	Narrative review	10.1016/j.trsl.2016.10.002	373	37.30	Q1
Liu S, 2020, Mol Neurobiol	Narrative review	10.1007/s12035-020-02073-3	356	50.86	Q1
Sandberg M, 2014, Neuropharmacology	Narrative review	10.1016/j.neuropharm.2013.11.004	347	26.69	Q1
Román GC, 2019, Rev Neurol-France	Narrative review	10.1016/j.neurol.2019.08.005	312	39.00	Q2
D’amico D, 2021, Trends Mol Med	Narrative review	10.1016/j.molmed.2021.04.009	290	48.33	Q1
Artmann A, 2008, BBA-Mol Cell Biol L	Animal study	10.1016/j.bbalip.2008.01.006	289	15.21	Q2
Mcgrattan AM, 2019, Curr Nutr Rep	Narrative review	10.1007/s13668-019-0271-4	287	35.88	Q1
Gubert C, 2020, Neurobiol Dis	Narrative review	10.1016/j.nbd.2019.104621	270	38.57	Q1
Bremner JD, 2020, Nutrients	Narrative review	10.3390/nu12082428	269	38.43	Q1
Solfrizzi v, 2017, j alzheimers dis	Systematic review	10.3233/JAD-170248	268	26.80	Q2
Dash S, 2015, Curr Opin Psychiatr	Narrative review	10.1097/YCO.0000000000000117	267	22.25	Q1
Adan Rah, 2019, Eur Neuropsychopharm	Narrative review	10.1016/j.euroneuro.2019.10.011	267	33.38	Q1
Berding K, 2021, Adv Nutr	Narrative review	10.1093/advances/nmaa181	256	42.67	Q1
Grosso G, 2014, Oxid Med Cell Longev	Narrative review	10.1155/2014/313570	249	19.15	Q1
Madison A, 2019, Curr Opin Behav Sci	Narrative review	10.1016/j.cobeha.2019.01.011	247	30.88	Q1

**Figure 7:**
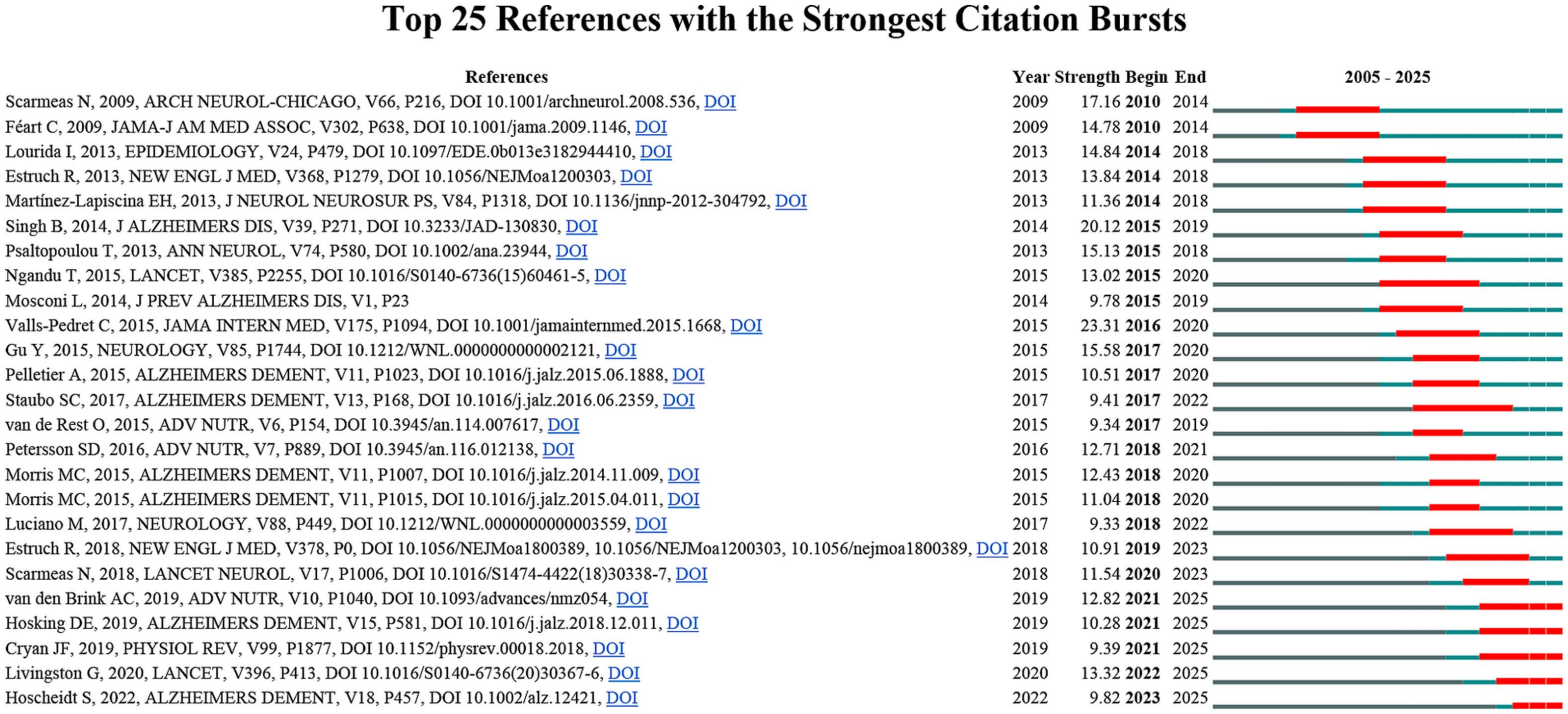
Top 25 references with the strongest citation bursts in MD in brain health.

To identify the most significant citation bursts related to MD in brain health, we used CiteSpace to obtain the top 25 articles with the strongest citation bursts (Figure 7). Among them, “Mediterranean Diet and Age-Related Cognitive Decline: A Randomized Clinical Trial” (burst strength: 23.31), “Association of Mediterranean diet with mild cognitive impairment and Alzheimer’s disease: a systematic review and meta-analysis” (burst strength: 20.12), and “Mediterranean diet and mild cognitive impairment” (burst strength: 17.16) exhibited the highest burst intensities. In addition, the three most recently published documents among the burst articles were: “Mediterranean and Western diet effects on Alzheimer’s disease biomarkers, cerebral perfusion, and cognition in mid-life: A randomized trial,” “Dementia prevention, intervention, and care: 2020 report of the Lancet Commission,” and “The Microbiota-Gut-Brain Axis.” To further understand the research frontiers and hotspots of MD in brain health, we matched the DOI of the 25 documents in Figure 7 with their titles. Analysis of these highly cited and burst documents indicates that, due to the growing attention to brain health, MD has gained widespread recognition as a potential dietary intervention. However, although it has shown benefits in the management of neurodegenerative diseases such as AD, long-term experimental data regarding its potential safety risks in other conditions, including depression, remain scarce. Therefore, this field warrants more in-depth research.

### 3.5 Keyword clusters and evolution

**Figure 8:**
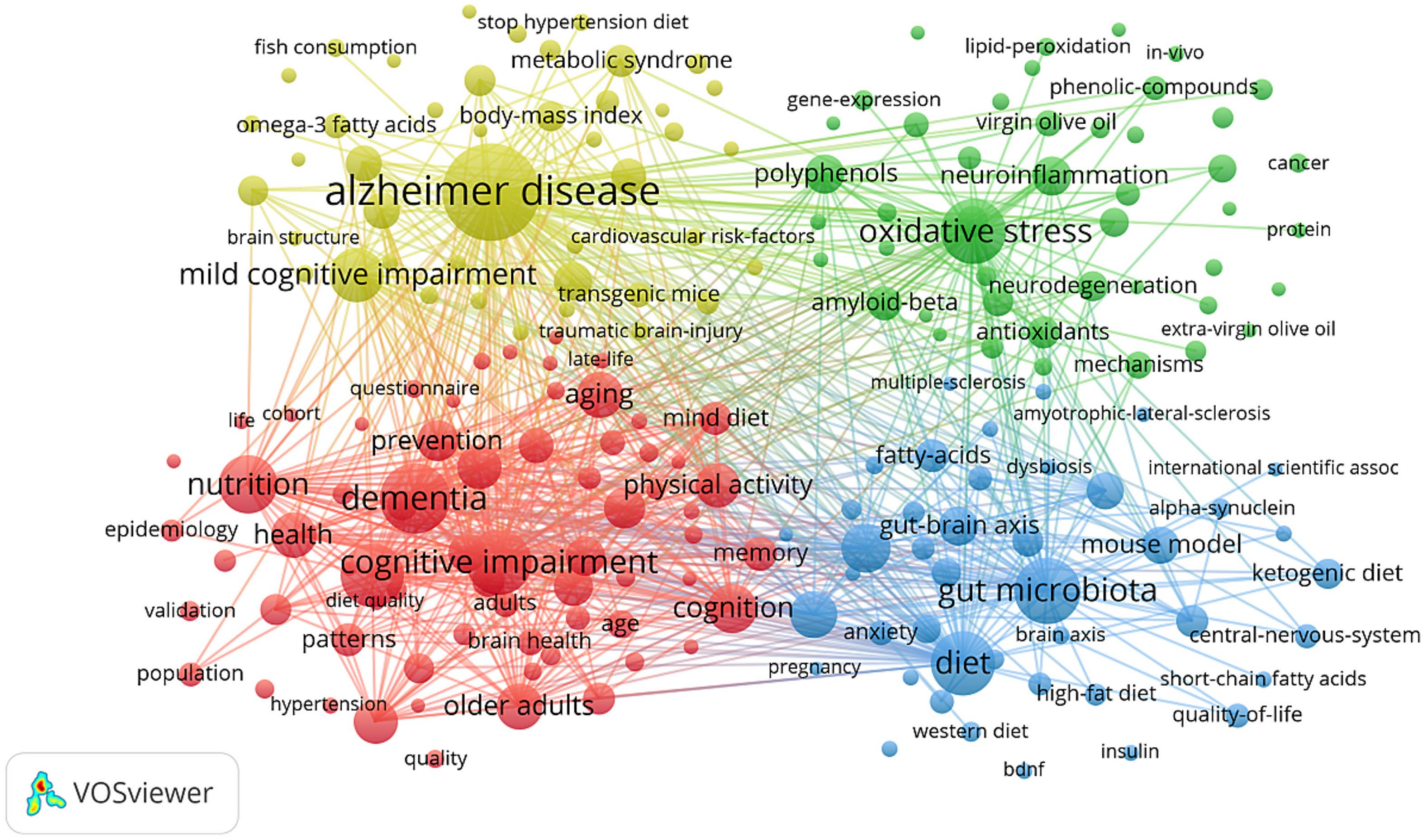
Keyword co-occurrence map of publications on MD in brain health.

Keyword clustering is an effective method for identifying research hotspots and developmental trends in a specific field. In this study, a total of 4,744 keywords were extracted using VOSviewer software. As shown in Table 8, the top 10 most frequent keywords each appeared ≥ 107 times. The most frequent keyword was Alzheimer disease (*n* = 400), followed by dementia (*n* = 214), gut microbiota (*n* = 192), cognitive impairment (*n* = 186), and oxidative stress (*n* = 186). Subsequently, 202 keywords were screened with a threshold of occurrence frequency ≥ 10 to construct a keyword clustering map (Figure 8). Four major research clusters were identified: The red cluster focused on the role of dietary patterns in cognitive decline and dementia prevention, including keywords such as dietary pattern, epidemiology, diet quality, validity, brain health, dementia prevention, neuroimaging, and hippocampus. The green cluster represented neuroprotection mediated by phenolic compounds and oxidative stress, with related keywords including oleuropein, phenolic compounds, resveratrol, oxidative stress, neuroinflammation, antioxidants, blood–brain barrier, brain-derived neurotrophic factor (BDNF), amyloid precursor protein, and neuroprotection. The yellow cluster addressed the effects of the MD on AD and vascular risk factors, including Alzheimer disease, cognitive impairment, elderly, polyunsaturated fatty acids, *ω*-3 fatty acids, vascular risk factors, cardiovascular disease, blood pressure, and risk factors. The blue cluster described the mechanisms by which the MD regulates neuropsychiatric disorders via the gut–brain axis, including gut microbiota, microbiota–gut–brain axis, metabolites, depression, PD, anxiety, mental health, and mechanism. VOSviewer was used to identify 196 Scopus keywords (Supplementary Figure S2). Current research on MD and brain health focuses on several key hotspots: (1) The intervention effects of dietary patterns on neurodegenerative diseases, particularly through modulation of the gut–brain axis; (2) the impact of MD on the aging brain and cognitive function; and (3) the neuroprotective effects and antioxidant mechanisms of vitamins, polyunsaturated fatty acids, and polyphenolic compounds.

**Table 8:** Top 10 keywords related to MD on brain health.

Rank	Keyword	Count
1	Alzheimer disease	400
2	dementia	214
3	gut microbiota	192
4	cognitive impairment	186
5	oxidative stress	186
6	diet	177
7	risk	172
8	nutrition	149
9	mild cognitive impairment	131
10	depression	107

**Figure 9:**
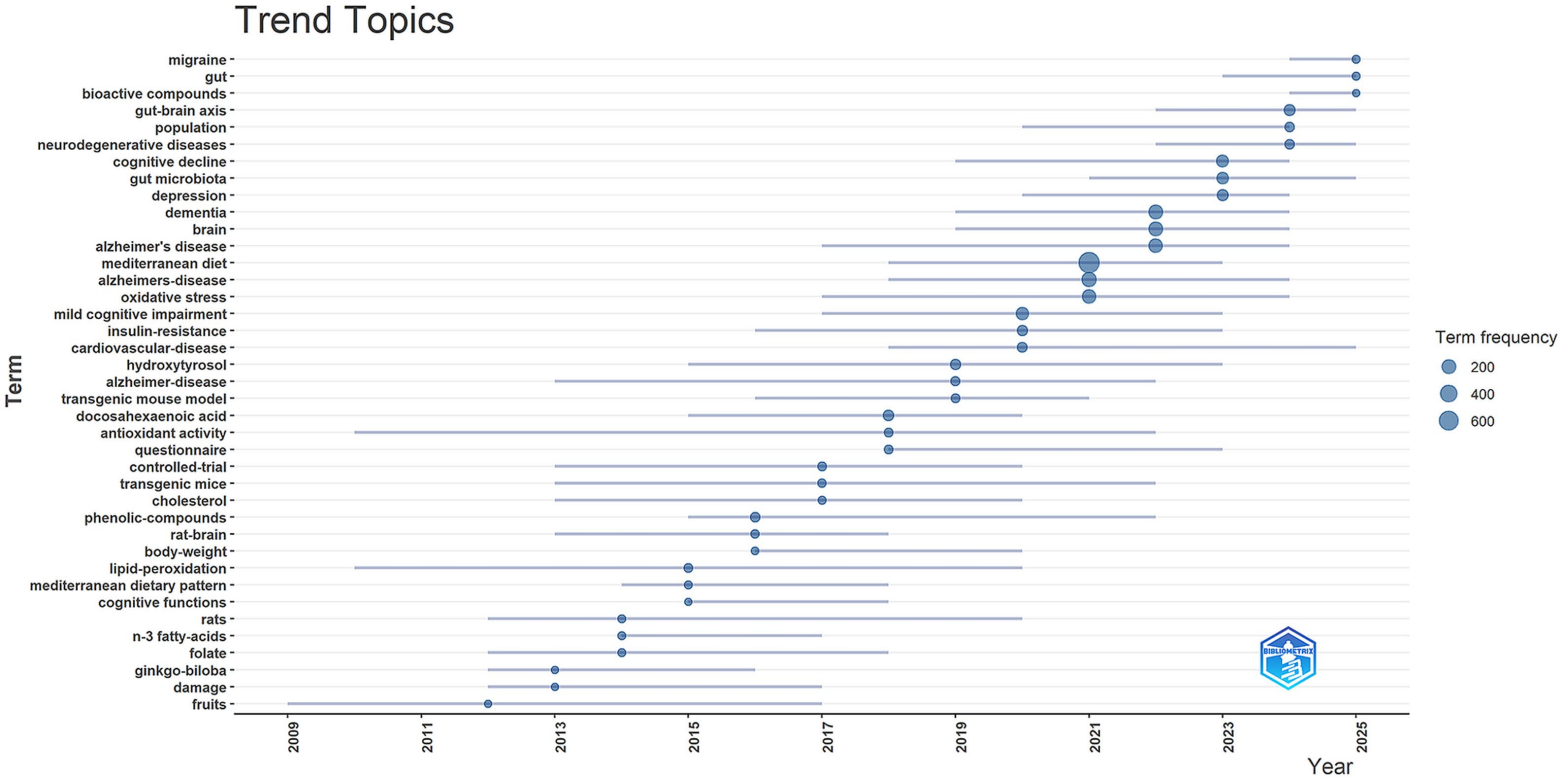
Trend topics in MD in brain health.

Theme trend analysis helps track the temporal evolution of specific research topics in a field, thereby capturing the changing patterns of research hotspots over time and facilitating a deeper understanding of their developmental trajectories. In this study, the Bibliometrix package in R was used to generate the thematic evolution trend map (Figure 9). The results showed that from 2009 to 2014, research focus was primarily on the antioxidant effects of specific nutrients or food components, and their neuroprotective effects were investigated using animal models. From 2015 to 2020, research gradually shifted toward clinical and epidemiological issues related to cognitive impairment and neurodegenerative diseases, with the “Mediterranean diet” emerging as a core theme as a holistic intervention model. From 2021 to 2025, research continued to focus on neurodegenerative diseases and depression, while the research frontier further expanded to cross-system mechanisms such as the “gut microbiota” and the “gut–brain axis,” reflecting the field’s shift toward multidimensional and integrative research directions.

## 4 Discussion

### 4.1 Basic information

To better understand the research hotspots and developmental trends of the MD in the field of brain health, we performed a bibliometric analysis and data visualization of 1,089 papers published between 2005 and 2025. The results showed a continuous upward trend in the number of relevant publications, which could be divided into a steady growth phase (before 2017) and a rapid growth phase (after 2017), indicating that research on MD and brain health has become a highly active topic. This trend may be closely associated with the launch of research projects on dietary patterns and brain health by multiple countries and institutions after 2017. For example, the US National Institutes of Health (NIH) initiated a multicultural healthy diet research program in 2017 (32), and the Australian National Health and Medical Research Council (NHMRC) launched the “Boosting Dementia Research Grants” special funding scheme (33). Furthermore, with the application of precision nutrition and multi-omics technologies, the field of MD and brain health is expected to remain a focus of intense research attention in the future.

#### 4.1.1 Countries/regions and institutions

The United States ranked first worldwide in the number of publications in this field, followed by Italy and Spain. The publication output of these three countries far exceeded that of other nations, indicating that researchers from these countries have shown strong interest in research related to MD and brain health and have invested substantial scientific resources. This highlights the regional characteristics of research in this field. The advantages of research institutions in European and American countries may be attributed to the fact that their traditional dietary patterns are more similar to the MD and that relevant research started earlier. An active collaborative network has been established among research institutions. In particular, Harvard University maintains close cooperation with top institutions such as Columbia University and the Instituto de Salud Carlos III in Spain. Professor Immaculata De Vivo from Harvard University, Professor Yian Gu from Columbia University, and Professor Marta Crous-Bou from the Instituto de Salud Carlos III collaborated and found through a meta-analysis that higher adherence to the MD was significantly associated with longer telomere length (34). Notably, Immaculata De Vivo is an internationally renowned scholar in molecular and genetic epidemiology and serves as Director of the Genotyping Facility at the Harvard Cancer Center. In addition, Professor Marta Crous-Bou is a highly active researcher on both sides of the Atlantic. She currently holds multiple research positions in Spain while maintaining long-term academic connections with Harvard University, playing an important role in promoting international collaboration.

#### 4.1.2 Journal analysis

The 1,089 papers were published in 415 academic journals. The main journals include *Nutrients*, *Journal of Alzheimer’s Disease*, *Neurology*, *American Journal of Clinical Nutrition*, and *Alzheimer’s & Dementia*. All these journals fall within the Q1–Q2 categories and have published influential studies on MD and brain health. In particular, *Nutrients* is both the most highly cited journal and the journal with the largest number of publications in this field. It is also ranked Q1 in the relevant JCR subject categories with a relatively high impact factor, indicating that it serves as the core publishing platform for research on MD and brain health. Meanwhile, several high-impact journals such as *Ageing Research Reviews* have published fewer articles but exhibit a higher citation rate per paper, which is closely related to their strict publication criteria and prominent academic influence. Articles published in these journals usually focus on cutting-edge, interdisciplinary topics with high innovation and theoretical depth. Therefore, despite their relatively small number, each article carries substantial academic value and has attracted extensive attention and frequent citations in subsequent studies. The high citation rate not only reflects the wide recognition of these research findings by the academic community but also demonstrates their key role in advancing the development of this field.

#### 4.1.3 Authors

Author and co-cited author analyses revealed extensive and close collaborations among researchers. Scarmeas N, Morris MC, and Jacka FN have exerted profound influences in this field. Among them, Scarmeas N made outstanding contributions. A representative work of Scarmeas N is a review published in *The Journal of the American Medical Association* in 2009. This review was the first to examine the association between the MD and the risk of cognitive decline and dementia in a European population. The results showed that higher adherence to the MD was significantly associated with slower global cognitive decline (35). In 2015, the team led by Scarmeas N analyzed magnetic resonance imaging (MRI) data from a community-based multi-ethnic elderly cohort and found that higher adherence to the MD was significantly associated with larger total brain volume, gray matter volume, and white matter volume (36). A 2022 study showed that specific nutrients abundant in the MD, such as antioxidant vitamins, polyphenols, and unsaturated fatty acids, were associated with better preservation of structural brain connectivity integrity and larger brain volume, thereby contributing to better brain health (37). A 2024 study found that higher adherence to the MD was significantly associated with enhanced negative connectivity between specific brain regions (e.g., temporo-occipital regions), and this connectivity pattern was positively correlated with better attention, executive function, and memory performance. These findings suggest that the MD may delay cognitive aging by optimizing the functional differentiation between the task-positive network and the default-mode network (38). Collectively, studies from Scarmeas N’s team have revealed the central protective role of the MD against cognitive aging. They have established a multi-dimensional evidence chain spanning from clinical cohorts to imaging mechanisms, laying a translational foundation for nutritional neuroscience from observational phenomena to mechanistic interpretations.

#### References

4.1.4

Highly cited articles reflect the most valuable and influential research findings and can further guide the research direction in a specific field. The paper *Diagnosis and Management of Dementia: Review*, published in *The Journal of the American Medical Association*, is the most highly cited article with a total of 931 citations. This article proposed that the diagnosis and management of dementia require comprehensive consideration of multiple neuropathological changes and multi-dimensional intervention strategies (39). The second-most cited study found that the MIND diet significantly slows cognitive decline in older adults, suggesting that an optimized dietary structure based on the MD is an effective strategy for delaying cognitive aging (40). The third-most cited article investigated the independent and combined effects of higher MD adherence and higher levels of physical activity on AD risk. It revealed that both factors independently reduce AD risk, and individuals who adhere to both a healthy diet and moderate exercise experience the most significant risk reduction, indicating a synergistic protective effect of a healthy lifestyle on brain health (41). Notably, all these highly cited papers fall within the Q1–Q2 journal tiers, demonstrating the high quality of foundational literature in this field.

Analysis of references with the strongest citation burst strength identifies the most influential publications in the field. The main citation burst period in this domain occurred between 2010 and 2023, during which a wealth of research emerged. This included studies on the impact of MD on brain aging and neurodegenerative diseases (42–44), as well as investigations into the roles of specific nutrients such as polyphenols, dietary fiber that promotes short-chain fatty acid production, and unsaturated fatty acids (45–47). The 2015 paper *Mediterranean diet and brain structure in a multiethnic elderly cohort*, published in *Neurology*, exhibited the strongest citation burst. This community-based, cross-sectional imaging study of a multiethnic elderly cohort found that higher MD adherence was significantly associated with larger total brain volume, gray matter volume, and white matter volume. The protective effect was equivalent to delaying brain aging by approximately 5 years, with high fish intake and low meat consumption identified as key contributing factors (36). The second-strongest citation burst was for a 2014 paper by Singh et al., *Association of Mediterranean diet with mild cognitive impairment and Alzheimer’s disease: a systematic review and meta-analysis*, published in *Journal of Alzheimer’s Disease*. This meta-analysis of five prospective cohort studies found that higher MD adherence was significantly associated with a reduced risk of mild cognitive impairment and AD, with a clear dose–response relationship (48). The third-strongest citation burst was for *Mediterranean diet and mild cognitive impairment*, published in *Archives of Neurology*. This study examined the impact of higher MD adherence on the risk of mild cognitive impairment and AD in older adults, revealing an inverse trend between MD adherence and the risk of incident mild cognitive impairment, as well as a significant reduction in the risk of progression from mild cognitive impairment to AD (49). Additionally, multiple papers currently in their citation burst phase indicate that the field has advanced into in-depth research on mechanisms related to the gut microbiota–gut–brain axis and the benefits of specific nutrients (46, 47).

### 4.2 Research hotspots and trends

Keyword analysis can highlight the core themes, research focuses and development trends of a research field, helping researchers grasp the knowledge framework and potential future research directions in this field. Through the analysis of citation frequency, citation bursts, keyword clustering and keyword trend topics in the field of MD and brain health research, the main focuses of current research can be clearly identified.

#### 4.2.1 Impact of the MD on brain aging

Keyword clustering, high-frequency keyword, and thematic trend analyses revealed that numerous terms related to brain aging frequently appeared, including brain health, aging, elderly, healthy aging, neuroimaging, hippocampus, brain volume, and functional connectivity. This indicates that the protective effects of MD in improving brain structure and delaying brain aging are the major focus of this field. In recent years, multiple human studies have confirmed that MD exerts significant protective effects in maintaining brain structure and optimizing brain function, providing a feasible dietary intervention strategy for delaying age-related brain aging. MD can increase brain volume and slow the progression of brain atrophy. An 18-month randomized controlled trial investigated the effects of the MD on brain health in 294 middle-aged and elderly participants with abdominal obesity/dyslipidemia. The results showed that adherence to the MD slowed the rate of hippocampal occupancy score decline by 50% and increased lateral ventricular volume (50). A cross-sectional study demonstrated that higher MD adherence was associated with reduced brain atrophy in older adults, an effect equivalent to slowing aging by 5 years (36). Neuroimaging evidence indicates that the MD is associated with increased cortical thickness in the frontal, parietal, and occipital lobes, enlarged dentate gyrus volume, and enhanced brain network connectivity (51, 52)—brain regions crucial for memory and emotional control. Moreover, the MD can slow cognitive decline and improve memory, attention, executive function, and overall cognitive performance (53). A meta-analysis including 19,734 participants showed that high MD adherence reduced the risk of global cognitive decline in non-demented older adults (54). A recent study by Yuan et al. revealed that the MD was associated with a reduced risk of incident dementia in middle-aged and elderly participants (55). Potential mechanisms include promotion of neurogenesis and synaptic plasticity, improvement of vascular risk factors such as blood pressure and blood glucose, inhibition of neuroinflammation and oxidative stress, and reduction of neuropathological protein load (56, 57).

Although these findings support the benefits of the MD in delaying brain aging, some studies have shown no statistically significant differences in brain MRI and cognitive outcomes between the MD and calorie-restricted control diets in healthy older adults (58). This negative result may be related to factors such as modifications in MD composition, study design, intervention duration, and ethnic differences. This highlights the importance of conducting further high-quality randomized controlled trials to verify the protective effects of the MD on brain aging. Additionally, efforts should be made to enrich MD intervention strategies; for example, combined MD and exercise interventions have shown better cognitive and metabolic improvements than the MD alone (59).

#### 4.2.2 Intervention effects of the MD on neuropsychiatric diseases

Through high-frequency keyword analysis, keyword clustering, and citation burst analysis, we found that AD, PD, and depression are important research topics in the field of the MD and brain health. The MD has potential application value in the prevention and treatment of the above major brain diseases. Its effects are related to multidimensional mechanisms, including reducing pathological protein load, inhibiting oxidative stress and neuroinflammation, regulating neurotransmitter levels, and improving cerebral perfusion and energy metabolism (60).

##### 4.2.2.1 Intervention effects of the MD on AD

AD is the most common neurodegenerative disorder of the central nervous system, characterized by progressive cognitive dysfunction and behavioral abnormalities. Compared with cognitively normal individuals, patients with AD show lower adherence to the MD (61). Adherence to the MD can significantly reduce the risk of AD (32) and delay disease progression by 1.5–3.5 years (62). The main pathological features of AD are the extracellular deposition of Aβ forming neuritic plaques and the intracellular accumulation of hyperphosphorylated tau protein (p-tau) forming neurofibrillary tangles, which lead to synaptic damage and neuronal degeneration. Studies have shown that high MD adherence is associated with reduced Aβ deposition in the brain (63). The MD can decrease cerebrospinal fluid Aβ deposition and tau phosphorylation in middle-aged and elderly subjects and improve pathological symptoms in AD models (24). This study also found that the MD increased cerebral blood flow (24). Enhanced cerebral perfusion not only improves hypoperfusion and hypoxia in brain tissue but also alleviates Aβ-induced neural damage, thereby slowing AD progression (64). In addition to Aβ and tau, mechanisms such as neuroinflammation, oxidative stress, and neurotransmitter imbalance are involved in AD pathogenesis and progression (65). The MD can improve insulin sensitivity, enhance brain glucose metabolism, reduce serum tumor necrosis factor-*α* and brain-derived neurotrophic factor(BDNF) expression, strengthen blood–brain barrier(BBB) integrity, and decrease oxidative stress, ultimately reducing the risk of AD progression (66, 67).

In summary, by reducing Aβ and tau deposition, improving cerebral perfusion and brain energy metabolism, alleviating neuroinflammation and oxidative stress, and increasing BDNF levels, the MD has emerged as a promising adjunctive treatment for the prevention and amelioration of AD. However, it should be noted that most existing clinical studies are observational, and the effects on patients with moderate-to-late AD remain unclear. In addition, poor long-term adherence is a major challenge for the MD in AD management. Further research in this field is needed to clarify the value of the MD in AD care.

##### 4.2.2.2 Intervention effects of the MD on PD

PD is a neurodegenerative disease characterized mainly by motor symptoms. Clinical evidence supports that the MD can be used in the intervention of PD, effectively reducing the risk of PD onset and improving cognitive function, motor ability, as well as non-motor symptoms such as constipation (68–71). Abnormal aggregation of *α*-synuclein is a typical pathological feature of PD, leading to progressive loss of dopaminergic neurons in the substantia nigra pars compacta and insufficient dopamine neurotransmission in the brain. In addition, α-synuclein accumulation can activate microglia to release inflammatory factors, triggering neuroinflammation and further neuronal damage, forming a vicious cycle (72). Studies have found that olive oil and its phenolic components can reduce *α*-synuclein aggregation in dopamine neurons, improve neuronal health, and enhance motor function (73), suggesting potential benefits in improving PD symptoms. The MD has also been shown to increase serum antioxidant capacity in PD patients, effectively scavenging excess free radicals, which may help reduce oxidative damage to neurons and alleviate disease severity (71). Furthermore, the MD can decrease fecal levels of zonulin and calprotectin—markers associated with intestinal permeability and inflammation—in PD patients (74). Improved intestinal barrier function reduces the entry of toxins and inflammatory factors into the bloodstream, alleviates neuroinflammation, and delays PD pathological progression (75).

In conclusion, the MD may exert neuroprotective effects in PD through antioxidant and anti-inflammatory regulation, modulation of the gut–brain axis, and reduction of abnormal α-synuclein aggregation and dopaminergic neuronal apoptosis, thus showing preliminary potential in PD intervention (76). However, PD patients often experience bradykinesia, rigidity, dysphagia, and other symptoms, making it difficult to ensure dietary adherence. At the same time, the optimal dosage and synergistic effects of specific nutritional components remain unclear. Individualized dietary guidelines should be developed to quickly match patients with appropriate dietary patterns and clarify the dose–response relationships between dietary components and PD prevention and control, providing a scientific basis for precise dietary interventions.

##### 4.2.2.3 Intervention effects of the MD on depression

Depression is a highly prevalent mental disorder worldwide, characterized by persistent low mood and diminished interest. The MD has significant potential in depression intervention, as it can regulate brain homeostasis through multiple pathways, alleviate depressive symptoms, and reduce disease risk (77–79). Studies have found that the MD can reduce white matter hyperintensity volume, which has a positive impact on improving depressive symptoms (60). Increased white matter hyperintensities are a recognized biomarker of depression. Reducing white matter hyperintensity levels helps improve structural connectivity in brain regions such as the frontal lobe, thalamus, and globus pallidus, enhancing attention, executive function, and information processing speed, and ultimately alleviating depressive mood (80). The MD also plays a key role in regulating chronic inflammation. Studies have shown that the MD can reduce serum pro-inflammatory cytokine levels, increase anti-inflammatory factors such as IL-10 and adiponectin, and inhibit the activation of inflammatory signaling pathways such as nuclear factor κB (NF-κB) (81, 82). By suppressing chronic inflammation, the MD helps improve brain neural circuits and synaptic connectivity, thereby alleviating depressive behaviors (27). In addition, obesity is a mediating factor in the association between the MD and depression (83). The MD can significantly improve metabolic health, which is crucial for depression. By stabilizing blood glucose levels, improving insulin sensitivity, and ameliorating dyslipidemia, the metabolic benefits of the MD may influence neuronal viability and neurotransmitter metabolism, thereby mitigating depressive progression (84).

In summary, by improving neuroinflammation and brain circuit connectivity, the MD helps regulate cognitive and emotional symptoms in depression patients and enhance mental health. By promoting metabolic homeostasis, this dietary pattern improves insulin sensitivity and lipid profiles, which may positively affect neuronal activity and neurotransmitter metabolism, making it a promising dietary therapy for depression. However, the clinical application of the MD should be based on individualized adjustments. Through the design of appropriate MD regimens and collaboration with medical experts and dietitians, the safety and sustainability of interventions can be ensured while maximizing brain health benefits.

#### 4.2.3 Mechanisms underlying the MD-mediated promotion of brain health

Nutrients such as polyphenolic compounds, dietary fiber, unsaturated fatty acids, vitamins, and minerals are the research focuses in this field, indicating that these nutritional components form the basis of the brain-protective effects exerted by the MD. Furthermore, gut microbiota has emerged as a research hotspot in recent years, suggesting that the MD targets gut microbiota as a core mediator for promoting brain health. Through multiple pathways including antioxidation, anti-inflammation, and neuroprotection, these nutrients ultimately confer benefits to brain health and cognitive function, and reduce the risk of developing neuropsychiatric disorders.

Polyphenolic compounds, widely present in whole grains, vegetables, fresh fruits, and nuts, exhibit powerful antioxidant, anti-inflammatory, and anti-apoptotic neuroprotective effects. For example, quercetin can activate nuclear factor erythroid 2-related factor 2 (Nrf2)/heme oxygenase-1 (HO-1) signaling pathway, reduce malondialdehyde(MDA), interleukin-1β (IL-1β), interleukin-6 (IL-6), and tumor necrosis factor-*α* (TNF-α) levels, alleviate oxidative stress and chronic inflammation in the brain, and delay aging-related brain atrophy (85). Resveratrol exerts neuroprotective effects by activating AMPK-related signaling pathways, inhibiting oxidative stress, reducing inflammatory cytokine release, suppressing neuronal apoptosis, and protecting synaptic function (86). Sirtuin 1(SIRT1) is an NAD + -dependent deacetylase that plays a central role in regulating cellular energy metabolism, stress resistance, and aging. Abnormal SIRT1 expression is closely associated with the pathogenesis of brain diseases (87). Polyphenolic compounds may regulate SIRT1 and its downstream pathways, including inhibition of neuroinflammation, enhancement of autophagy, and restoration of mitochondrial function, while synergizing with signaling axes such as 5′ adenosine monophosphate-activated protein kinase (AMPK), NF-κB, Nrf2, and peroxisome proliferator-activated receptor gamma coactivator 1-alpha (PGC-1α) to promote brain health (88). In addition, polyphenols have been shown to affect the synthesis and metabolism of neurotransmitters such as serotonin and dopamine and inhibit the abnormal aggregation of Aβ, thereby exerting positive effects on mood and cognitive function (89, 90).

Olive oil is rich in fatty acids beneficial to metabolic health, including monounsaturated fatty acids (MUFAs) and polyunsaturated fatty acids (PUFAs). MUFAs improve brain health through the following mechanisms: first, by increasing GM1 ganglioside content in lipid rafts, enhancing synaptic plasticity, regulating ion channel activity, and improving the fluidity and stability of neuronal membranes to ensure normal neural signal transmission (91, 92); second, by activating tyrosine kinase receptor B (TRKB) and increasing BDNF secretion; third, by regulating lipid metabolism, protecting vascular endothelial function, and improving cerebral blood circulation (93). PUFAs promote the proliferation and differentiation of neural stem cells, increase the generation of new neurons, and contribute to brain repair and regeneration (94). They also regulate glutamate receptors on the postsynaptic membrane, promoting the formation of long-term potentiation and thus improving cognitive function (95). In addition, PUFAs have vascular protective effects. Omega-3 polyunsaturated fatty acids can stimulate the expression of angiogenic factors such as vascular endothelial growth factor (VEGF), promote cerebrovascular formation and repair, and indirectly protect brain health by reducing blood pressure (96, 97).

The abundant vitamins A, C, D, and E in the MD are also important nutritional components. Vitamin C is a potent antioxidant that directly scavenges reactive oxygen species (ROS) and forms a synergistic antioxidant network with vitamin E to protect neurons from oxidative damage (98). It also participates in the regulation of neurotransmitter metabolism and neuronal development and maturation, thereby maintaining brain health (99). Vitamin A can activate retinoic acid receptors (RARs), upregulate the expression of ferroptosis-resistant genes, and inhibit lipid peroxidation, thereby preventing neuronal ferroptosis and maintaining neuronal survival and function (100). Vitamin D acts on signaling pathways such as Nrf2, BDNF, Wnt/*β*-catenin, and Toll-like receptor 4/NF-κB, exerting neuroprotective effects by reducing oxidative stress, promoting neurogenesis and synaptic plasticity, regulating neuroinflammation and immune homeostasis, and delaying cellular senescence, thus lowering the risk of brain diseases (101, 102). In addition, the intake of potassium, calcium, and magnesium in the MD helps reduce peripheral vascular resistance, promote vascular smooth muscle relaxation, and control blood pressure, ultimately benefiting cerebrovascular health (103, 104).

Notably, recent studies have found a close association between the MD, the gut–brain axis, and neuroprotection. Dietary fiber, polyphenols, and other components abundant in the MD can increase the abundance of beneficial gut bacteria (such as Bifidobacterium and *Faecalibacterium prausnitzii*), reduce harmful bacteria (such as Ruminococcus), and maintain gut microbial balance (105, 106). Short-chain fatty acids produced by gut microbiota through fermentation of dietary fiber can not only affect neurotransmitter secretion and neuroplasticity in the brain via the vagus nerve but also enter the bloodstream, act on the brain, and regulate physiological processes such as energy metabolism and antioxidant defense, thereby enhancing neuronal survival and function (107). In addition, a healthy gut microbiota can inhibit inflammatory responses and prevent neuroinflammatory damage to the brain (108). Meanwhile, amino acids such as tryptophan in the diet can be converted into neurotransmitters such as serotonin, which influence brain function through the gut–brain axis, thereby improving mood and cognitive ability (109). Therefore, the MD may regulate gut microbiota, metabolites, and neurotransmitters to activate the neuroprotective mechanisms of the gut–brain axis, thereby promoting brain health.

However, through literature analysis, it can be seen that the specific mechanisms by which the MD promotes brain health, especially in the intervention of neuropsychiatric diseases, have not been fully elucidated. The synergistic, interactive, or antagonistic effects among various nutritional components, as well as the mechanisms through which the MD exerts neuroprotection via the gut microbiota–gut–brain axis, still require further exploration (110). Therefore, future research should identify the key targets of various dietary components on neuronal signaling networks; particularly important is to clarify the role of gut microbiota and their metabolites. In addition, it is necessary to investigate the heterogeneous responses of different disease populations (such as those with depression, AD, and PD) to dietary components and explore personalized dietary intervention strategies to provide more precise nutritional support for neuroprotection.

### 4.3 Limitations

This study aims to improve the understanding of the current status and research hotspots of MD in the field of brain health, and to explore valuable potential research directions. However, this study still has several limitations. First, this study only included data from the WoSCC and Scopus databases, which may lead to the omission of literature published exclusively in other databases. Nevertheless, WoSCC and Scopus are widely recognized for their high quality and credibility, making them reasonable data sources for the bibliometric analysis in this study. Second, only English-language literature was analyzed in this study, which may neglect research conducted in other languages and introduce data source bias. Third, in terms of document types, only reviews and original articles were included, which may also result in data omission. In addition, the core authors identified in this study showed a certain degree of team clustering. Some high-productivity authors belonged to the same laboratory or research group, which may bias the assessment of their influence in the field and underestimate the academic contributions of independent researchers. Despite these limitations, this study provides a comprehensive overview of the field and clarifies its core themes and developmental trends. By considering the above factors, researchers can gain a deeper insight into the overall progress of the field, identify potential research directions, and provide references and guidance for future investigations.

## 5 Conclusion

Our study clearly identifies the key research hotspots and emerging frontiers of the MD in the field of brain health. The following is a summary of the main knowledge directions and research trends in this field:

a MD in brain health has attracted global research attention, with countries such as the United States, Italy, and Spain serving as core research forces, demonstrating high research activity and close international collaboration. As research on MD and brain health continues to deepen in the future, cooperation among countries will further strengthen.
b In this research field, the journal *Nutrients* ranks first in both the number of publications and citations, fully reflecting its representative status as a core academic platform in the field.
c Co-author analysis of MD and brain health research reveals extensive and close collaborations among authors, with Scarmeas N, Aggarwal Nt, and Barnes Ll as representative contributors.
d The impact of MD on brain aging has become a major hotspot and trend in brain health research.
e The intervention effects of MD on AD, PD, and depression have emerged as key hotspots and trends in brain health research.
f Mechanistic studies on how MD promotes brain health have focused on the antioxidant, anti-inflammatory, and neuroprotective effects of multiple core nutritional components, and gut microbiota regulation has gradually become an emerging hotspot in mechanistic research.

In conclusion, our study provides valuable insights into the research trends and hotspots of MD in the field of brain health. These findings help researchers quickly grasp the current status of the field and identify new directions for future exploration. By pointing out the limitations of current research and potential key areas, this study offers guidance for researchers to deepen related investigations and encourages them to develop innovative research pathways in their work.

## Data Availability

The datasets presented in this study can be found in online repositories. The names of the repository/repositories and accession number(s) can be found in the article/Supplementary material.

## References

[1] Feng S Wang T Su Y Yan J Wang Y Zhang Z . Global burden, risk factors, and projections of early-onset dementia: insights from the global burden of disease study 2021. Ageing Res Rev. (2025) 104:102644. doi: 10.1016/j.arr.2024.102644, 39701185

[2] Khemka S Reddy A Garcia RI Jacobs M Reddy RP Roghani AK . Role of diet and exercise in aging, alzheimer's disease, and other chronic diseases. Ageing Res Rev. (2023) 91:102091. doi: 10.1016/j.arr.2023.102091, 37832608 PMC10842571

[3] China KBCG. Joint impact of polygenic risk score and lifestyles on early- and late-onset cardiovascular diseases. Nat Hum Behav. (2024) 8:1810–8. doi: 10.1038/s41562-024-01923-7, 38987358

[4] Cao Z Yang H Ye Y Zhang Y Li S Zhao H . Polygenic risk score, healthy lifestyles, and risk of incident depression. Transl Psychiatry. (2021) 11:189. doi: 10.1038/s41398-021-01306-w, 33782378 PMC8007584

[5] Santisteban MM Iadecola C. The pathobiology of neurovascular aging. Neuron. (2025) 113:49–70. doi: 10.1016/j.neuron.2024.12.014, 39788087 PMC12136575

[6] Jin S Lu W Zhang J Zhang L Tao F Zhang Y . The mechanisms, hallmarks, and therapies for brain aging and age-related dementia. Sci Bull (Beijing). (2024) 69:3756–76. doi: 10.1016/j.scib.2024.09.005, 39332926

[7] Chu M Jiang D Li D Yan S Liu L Nan H . Atrophy network mapping of clinical subtypes and main symptoms in frontotemporal dementia. Brain J Neurol. (2024) 147:3048–58. doi: 10.1093/brain/awae067, 38426222 PMC11370799

[8] Mintzer J Donovan KA Kindy AZ Lock SL Chura LR Barracca N. Lifestyle choices and brain health. Front Med (Lausanne). (2019) 6:204. doi: 10.3389/fmed.2019.00204, 31637242 PMC6787147

[9] Rajaram S Jones J Lee GJ. Plant-based dietary patterns, plant foods, and age-related cognitive decline. Adv Nutr (Bethesda, Md). (2019) 10:S422–36. doi: 10.1093/advances/nmz081, 31728502 PMC6855948

[10] Boa Sorte Silva NC Barha CK Erickson KI Kramer AF Liu-Ambrose T. Physical exercise, cognition, and brain health in aging. Trends Neurosci. (2024) 47:402–17. doi: 10.1016/j.tins.2024.04.004, 38811309

[11] Pérez Palmer N Trejo Ortega B Joshi P. Cognitive impairment in older adults: epidemiology, diagnosis, and treatment. Psychiatr Clin North Am. (2022) 45:639–61. doi: 10.1016/j.psc.2022.07.010, 36396270

[12] Villéga F Fernandes A Jézéquel J Uyttersprot F Benac N Zenagui S . Ketamine alleviates nmda receptor hypofunction through synaptic trapping. Neuron. (2024) 112:3311–3328.e9. doi: 10.1016/j.neuron.2024.06.028, 39047728

[13] Atkin T Comai S Gobbi G. Drugs for insomnia beyond benzodiazepines: pharmacology, clinical applications, and discovery. Pharmacol Rev. (2018) 70:197–245. doi: 10.1124/pr.117.014381, 29487083

[14] Heneka MT Morgan D Jessen F. Passive anti-amyloid β immunotherapy in Alzheimer's disease-opportunities and challenges. Lancet (London, England). (2024) 404:2198–208. doi: 10.1016/S0140-6736(24)01883-X, 39549715

[15] Moussavi Z Uehara M Rutherford G Lithgow B Millikin C Wang X . Repetitive transcranial magnetic stimulation as a treatment for Alzheimer's disease: a randomized placebo-controlled double-blind clinical trial. Neurotherapeutics. (2024) 21:e331. doi: 10.1016/j.neurot.2024.e00331, 38360452 PMC10937236

[16] Kamel WA Al-Hashel JY. Lcig in treatment of non-motor symptoms in advanced Parkinson's disease: review of literature. Brain Behav. (2020) 10:e1757. doi: 10.1002/brb3.1757, 32677345 PMC7507541

[17] Smit JW Basile P Prato MK Detalle L Mathy FO Schmidt A . Phase 1/1b studies of ucb0599, an oral inhibitor of α-synuclein misfolding, including a randomized study in Parkinson's disease. Mov Disord. (2022) 37:2045–56. doi: 10.1002/mds.29170, 35959805 PMC9804489

[18] Schramm E Klein DN Elsaesser M Furukawa TA Domschke K. Review of dysthymia and persistent depressive disorder: history, correlates, and clinical implications. Lancet Psychiatry. (2020) 7:801–12. doi: 10.1016/S2215-0366(20)30099-7, 32828168

[19] Kovich H Kim W Quaste AM. Pharmacologic treatment of depression. Am Fam Physician. (2023) 107:173–81. 36791444

[20] Cuijpers P Karyotaki E Eckshtain D Ng MY Corteselli KA Noma H . Psychotherapy for depression across different age groups: a systematic review and meta-analysis. JAMA Psychiatry. (2020) 77:694–702. doi: 10.1001/jamapsychiatry.2020.0164, 32186668 PMC7081149

[21] Akerele CA Koralnik LR Lafont E Gilman C Walsh-Messinger J Malaspina D. Nutrition and brain health: implications of mediterranean diet elements for psychiatric disorders. Schizophr Res. (2025) 281:30–44. doi: 10.1016/j.schres.2025.04.026, 40315757

[22] Wi Niewska K Okr Glicka KMG Paskudzka M Jagielska AM Bober J Oczkowski M . The vegprev study: effectiveness of four plant-based diets on weight loss, metabolic syndrome components and appetitive traits in overweight and obese individuals: a randomized controlled trial. Front Nutr. (2025) 12:1677496. doi: 10.3389/fnut.2025.1677496 41340660 PMC12668910

[23] Arjunan A Sah DK Woo M Song J. Identification of the molecular mechanism of insulin-like growth factor-1 (igf-1): a promising therapeutic target for neurodegenerative diseases associated with metabolic syndrome. Cell Biosci. (2023) 13:16. doi: 10.1186/s13578-023-00966-z, 36691085 PMC9872444

[24] Hoscheidt S Sanderlin AH Baker LD Jung Y Lockhart S Kellar D . Mediterranean and western diet effects on alzheimer's disease biomarkers, cerebral perfusion, and cognition in mid-life: a randomized trial. Alzheimers Dement. (2022) 18:457–68. doi: 10.1002/alz.12421, 34310044 PMC9207984

[25] Weijs RWJ Shkredova DA Brekelmans ACM Thijssen DHJ Claassen JAHR. Longitudinal changes in cerebral blood flow and their relation with cognitive decline in patients with dementia: current knowledge and future directions. Alzheimers Dement. (2023) 19:532–48. doi: 10.1002/alz.12666, 35485906

[26] Staudacher HM Teasdale S Cowan C Opie R Jacka FN Rocks T. Diet interventions for depression: review and recommendations for practice. Aust N Z J Psychiatry. (2025) 59:115–27. doi: 10.1177/00048674241289010, 39628343 PMC11783990

[27] Kurowska A Ziemichód W Herbet M Pi Tkowska-Chmiel I. The role of diet as a modulator of the inflammatory process in the neurological diseases. Nutrients. (2023) 15:1436. doi: 10.3390/nu15061436, 36986165 PMC10057655

[28] Cardelo MP Corina A Leon-Acu A A Quintana-Navarro GM Alcala-Diaz JF Rangel-Zu Iga OA . Effect of the mediterranean diet and probiotic supplementation in the management of mild cognitive impairment: rationale, methods, and baseline characteristics. Front Nutr. (2022) 9:1037842. doi: 10.3389/fnut.2022.1037842 36570150 PMC9773830

[29] Meng Y Tian J Xiu Li X Xu Z. Associations of mind and di-gm dietary scores with depression, anxiety, and gut microbiota in patients with colon cancer: a cross-sectional study. Front Nutr. (2025) 12:1655051. doi: 10.3389/fnut.2025.1655051, 40927565 PMC12414760

[30] Tian S Chen M. Global research progress of gut microbiota and epigenetics: bibliometrics and visualized analysis. Front Immunol. (2024) 15:1412640. doi: 10.3389/fimmu.2024.1412640, 38803501 PMC11128553

[31] Wang Y Liu T Liu W Zhao H Li P. Research hotspots and future trends in lipid metabolism in chronic kidney disease: a bibliometric and visualization analysis from 2004 to 2023. Front Pharmacol. (2024) 15:1401939. doi: 10.3389/fphar.2024.1401939, 39290864 PMC11405329

[32] Liu Y Gu X Li Y Wang F Vyas CM Peng C . Interplay of genetic predisposition, plasma metabolome and mediterranean diet in dementia risk and cognitive function. Nat Med. (2025) 31:3790–800. doi: 10.1038/s41591-025-03891-5, 40855194 PMC12618253

[33] Bracci EL Davis CR Meyer D Kingsley M Breckon J Minihane A . The cost of a mediterranean diet and walking intervention to reduce risk of dementia and improve quality of life in independent living populations in Australia: the medwalk randomised controlled trial. Br J Nutr. (2025) 134:969–78. doi: 10.1017/S0007114525105333, 41102970

[34] Canudas S Becerra-Tomás N Hernández-Alonso P Galié S Leung C Crous-Bou M . Mediterranean diet and telomere length: a systematic review and meta-analysis. Adv Nutr. (2020) 11:1544–54. doi: 10.1093/advances/nmaa079, 32730558 PMC7666892

[35] Féart C Samieri C Rondeau V Amieva H Portet F Dartigues JO . Adherence to a mediterranean diet, cognitive decline, and risk of dementia. JAMA. (2009) 302:638–48. doi: 10.1001/jama.2009.1146, 19671905 PMC2850376

[36] Gu Y Brickman AM Stern Y Habeck CG Razlighi QR Luchsinger JA . Mediterranean diet and brain structure in a multiethnic elderly cohort. Neurology. (2015) 85:1744–51. doi: 10.1212/WNL.0000000000002121, 26491085 PMC4653103

[37] Drouka A Mamalaki E Karavasilis E Scarmeas N Yannakoulia M. Dietary and nutrient patterns and brain mri biomarkers in dementia-free adults. Nutrients. (2022) 14:2345. doi: 10.3390/nu14112345, 35684145 PMC9183163

[38] Karavasilis E Balomenos V Christidi F Velonakis G Angelopoulou G Yannakoulia M . Mediterranean diet and brain functional connectivity in a population without dementia. Front Neuroimag. (2024) 3:1473399. doi: 10.3389/fnimg.2024.1473399, 39713787 PMC11659224

[39] Arvanitakis Z Shah RC Bennett DA. Diagnosis and management of dementia: review. JAMA. (2019) 322:1589–99. doi: 10.1001/jama.2019.4782, 31638686 PMC7462122

[40] Morris MC Tangney CC Wang Y Sacks FM Barnes LL Bennett DA . Mind diet slows cognitive decline with aging. Alzheimers Dement. (2015) 11:1015–22. doi: 10.1016/j.jalz.2015.04.011, 26086182 PMC4581900

[41] Scarmeas N Luchsinger JA Schupf N Brickman AM Cosentino S Tang MX . Physical activity, diet, and risk of alzheimer disease. JAMA. (2009) 302:627–37. doi: 10.1001/jama.2009.1144, 19671904 PMC2765045

[42] Lourida I Soni M Thompson-Coon J Purandare N Lang IA Ukoumunne OC . Mediterranean diet, cognitive function, and dementia: a systematic review. Epidemiology. (2013) 24:479–89. doi: 10.1097/EDE.0b013e3182944410, 23680940

[43] Martínez-Lapiscina EH Clavero P Toledo E Estruch R Salas-Salvadó J San Julián B . Mediterranean diet improves cognition: the predimed-Navarra randomised trial. J Neurol Neurosurg Psychiatry. (2013) 84:1318–25. doi: 10.1136/jnnp-2012-304792, 23670794

[44] Ngandu T Lehtisalo J Solomon A Lev Lahti E Ahtiluoto S Antikainen R . A 2 year multidomain intervention of diet, exercise, cognitive training, and vascular risk monitoring versus control to prevent cognitive decline in at-risk elderly people (finger): a randomised controlled trial. Lancet (London, England). (2015) 385:2255–63. doi: 10.1016/S0140-6736(15)60461-5 25771249

[45] Scarmeas N Anastasiou CA Yannakoulia M. Nutrition and prevention of cognitive impairment. Lancet Neurol. (2018) 17:1006–15. doi: 10.1016/S1474-4422(18)30338-7, 30244829

[46] van den Brink AC Brouwer-Brolsma EM Berendsen AAM van de Rest O. The mediterranean, dietary approaches to stop hypertension (dash), and mediterranean-dash intervention for neurodegenerative delay (mind) diets are associated with less cognitive decline and a lower risk of Alzheimer's disease-a review. Adv Nutr. (2019) 10:1040–65. doi: 10.1093/advances/nmz054, 31209456 PMC6855954

[47] Cryan JF O'Riordan KJ Cowan CSM Sandhu KV Bastiaanssen TFS Boehme M . The microbiota-gut-brain axis. Physiol Rev. (2019) 99:1877–2013. doi: 10.1152/physrev.00018.2018, 31460832

[48] Singh B Parsaik AK Mielke MM Erwin PJ Knopman DS Petersen RC . Association of mediterranean diet with mild cognitive impairment and alzheimer's disease: a systematic review and meta-analysis. J Alzheimer's Dis: Jad. (2014) 39:271–82. doi: 10.3233/JAD-130830, 24164735 PMC3946820

[49] Scarmeas N Stern Y Mayeux R Manly JJ Schupf N Luchsinger JA. Mediterranean diet and mild cognitive impairment. Arch Neurol. (2009) 66:216–25. doi: 10.1001/archneurol.2008.536, 19204158 PMC2653223

[50] Kaplan A Zelicha H Yaskolka Meir A Rinott E Tsaban G Levakov G . The effect of a high-polyphenol mediterranean diet (green-med) combined with physical activity on age-related brain atrophy: the dietary intervention randomized controlled trial polyphenols unprocessed study (direct plus). Am J Clin Nutr. (2022) 115:1270–81. doi: 10.1093/ajcn/nqac001, 35021194 PMC9071484

[51] Staubo SC Aakre JA Vemuri P Syrjanen JA Mielke MM Geda YE . Mediterranean diet, micronutrients and macronutrients, and mri measures of cortical thickness. Alzheimers Dement. (2017) 13:168–77. doi: 10.1016/j.jalz.2016.06.2359, 27461490 PMC5259552

[52] Karstens AJ Tussing-Humphreys L Zhan L Rajendran N Cohen J Dion C . Associations of the mediterranean diet with cognitive and neuroimaging phenotypes of dementia in healthy older adults. Am J Clin Nutr. (2019) 109:361–8. doi: 10.1093/ajcn/nqy275, 30698630 PMC6367961

[53] Papandreou C Papagiannopoulos C Koutsonida M Kanellopoulou A Markozannes G Polychronidis G . Mediterranean diet related metabolite profiles and cognitive performance. Clinical Nutrition (Edinburgh, Scotland). (2023) 42:173–81. doi: 10.1016/j.clnu.2022.12.012, 36599272

[54] Coelho-Júnior HJ Trichopoulou A Panza F. Cross-sectional and longitudinal associations between adherence to mediterranean diet with physical performance and cognitive function in older adults: a systematic review and meta-analysis. Ageing Res Rev. (2021) 70:101395. doi: 10.1016/j.arr.2021.101395, 34153553

[55] Chen H Dhana K Huang Y Huang L Tao Y Liu X . Association of the mediterranean dietary approaches to stop hypertension intervention for neurodegenerative delay (mind) diet with the risk of dementia. JAMA Psychiatr. (2023) 80:630–8. doi: 10.1001/jamapsychiatry.2023.0800, 37133875 PMC10157510

[56] Tsolaki M Lazarou E Kozori M Petridou N Tabakis I Lazarou I . A randomized clinical trial of Greek high phenolic early harvest extra virgin olive oil in mild cognitive impairment: the micoil pilot study. J Alzheimer's Dis. (2020) 78:801–17. doi: 10.3233/JAD-200405, 33044178

[57] Tuttolomondo A Simonetta I Daidone M Mogavero A Ortello A Pinto A. Metabolic and vascular effect of the mediterranean diet. Int J Mol Sci. (2019) 20:4716. doi: 10.3390/ijms20194716, 31547615 PMC6801699

[58] Barnes LL Dhana K Liu X Carey VJ Ventrelle J Johnson K . Trial of the mind diet for prevention of cognitive decline in older persons. N Engl J Med. (2023) 389:602–11. doi: 10.1056/NEJMoa2302368, 37466280 PMC10513737

[59] Ward NA Reid-McCann R Brennan L Cardwell CR de Groot C Maggi S . Effects of protein enriched mediterranean diet and exercise on nutritional status and cognition in adults at risk of undernutrition and cognitive decline: the promed-ex randomised controlled trial. BMJ Open. (2023) 13:e70689. doi: 10.1136/bmjopen-2022-070689, 37880167 PMC10603411

[60] Shi Y Lin Y Zheng Y Yu X Ou B Yang K . Association between different dietary patterns and the risk of major brain disorders: a prospective multi-cohort study. EClinicalMedicine. (2025) 90:103616. doi: 10.1016/j.eclinm.2025.103616, 41245532 PMC12615346

[61] Wu L Sun D. Adherence to mediterranean diet and risk of developing cognitive disorders: an updated systematic review and meta-analysis of prospective cohort studies. Sci Rep. (2017) 7:41317. doi: 10.1038/srep41317, 28112268 PMC5256032

[62] Berti V Walters M Sterling J Quinn CG Logue M Andrews R . Mediterranean diet and 3-year alzheimer brain biomarker changes in middle-aged adults. Neurology. (2018) 90:e1789–98. doi: 10.1212/WNL.0000000000005527, 29653991 PMC5957301

[63] Rainey-Smith SR Gu Y Gardener SL Doecke JD Villemagne VL Brown BM . Mediterranean diet adherence and rate of cerebral aβ-amyloid accumulation: data from the australian imaging, biomarkers and lifestyle study of ageing. Transl Psychiatry. (2018) 8:238. doi: 10.1038/s41398-018-0293-5, 30375373 PMC6207555

[64] Bragina OA Sillerud LO Kameneva MV Nemoto EM Bragin DE. Haemorheologic enhancement of cerebral perfusion improves oxygen supply and reduces aβ plaques deposition in a mouse model of alzheimer's disease. Adv Exp Med Biol. (2022) 1395:335–40. doi: 10.1007/978-3-031-14190-4_54, 36527658 PMC10036199

[65] Wei L Li Z Shi M Song W Teng Z Zhang C. Neuroprotective properties of extra virgin olive oil polyphenols in alzheimer's disease: a multi-target mechanistic review. Front Nutr. (2025) 12:1736633. doi: 10.3389/fnut.2025.1736633, 41393957 PMC12695547

[66] Visioli F Rodríguez-Pérez M Gómez-Torres Ó Pintado-Losa C Burgos-Ramos E. Hydroxytyrosol improves mitochondrial energetics of a cellular model of alzheimer's disease. Nutr Neurosci. (2022) 25:990–1000. doi: 10.1080/1028415X.2020.1829344, 33023416

[67] Alkhalifa AE Al-Ghraiybah NF Kaddoumi A. Extra-virgin olive oil in alzheimer's disease: a comprehensive review of cellular, animal, and clinical studies. Int J Mol Sci. (2024) 25:1914. doi: 10.3390/ijms25031914, 38339193 PMC10856527

[68] Hajji-Louati M Correia E Lee P Artaud F Roze E Mancini FR . Adherence to the mediterranean and mediterranean-dietary approaches to stop hypertension intervention for neurodegenerative delay (mind) diets and Parkinson's disease incidence in women: results from the prospective e3n cohort. Ann Neurol. (2026) 16:10–1002. doi: 10.1002/ana.78115 PMC13011782 41492937

[69] Zhao J Peng Y Lin Z Gong Y. Association between mediterranean diet adherence and Parkinson's disease: a systematic review and meta-analysis. J Nutr Health Aging. (2025) 29:100451. doi: 10.1016/j.jnha.2024.100451, 39693849 PMC12180059

[70] Paknahad Z Sheklabadi E Derakhshan Y Bagherniya M Chitsaz A. The effect of the Mediterranean diet on cognitive function in patients with Parkinson's disease: a randomized clinical controlled trial. Complement Ther Med. (2020) 50:102366. doi: 10.1016/j.ctim.2020.102366, 32444045

[71] Paknahad Z Sheklabadi E Moravejolahkami AR Chitsaz A Hassanzadeh A. The effects of mediterranean diet on severity of disease and serum total antioxidant capacity (tac) in patients with parkinson's disease: a single center, randomized controlled trial. Nutr Neurosci. (2022) 25:313–20. doi: 10.1080/1028415X.2020.1751509, 32319358

[72] Yuan X Nie S Yang Y Liu C Xia D Meng L . Propagation of pathologic α-synuclein from kidney to brain may contribute to parkinson's disease. Nat Neurosci. (2025) 28:577–88. doi: 10.1038/s41593-024-01866-2, 39849144

[73] Di Rosa G Brunetti G Scuto M Trovato Salinaro A Calabrese EJ Crea R . Healthspan enhancement by olive polyphenols in *c. Elegans* wild type and Parkinson's models. Int J Mol Sci. (2020) 21:3893. doi: 10.3390/ijms21113893, 32486023 PMC7312680

[74] Rusch C Beke M Tucciarone L Nieves CJ Ukhanova M Tagliamonte MS . Mediterranean diet adherence in people with Parkinson's disease reduces constipation symptoms and changes fecal microbiota after a 5-week single-arm pilot study. Front Neurol. (2021) 12:794640. doi: 10.3389/fneur.2021.794640, 35002935 PMC8733603

[75] Ayten E Bilici S. Modulation of gut microbiota through dietary intervention in neuroinflammation and alzheimer's and parkinson's diseases. Curr Nutr Rep. (2024) 13:82–96. doi: 10.1007/s13668-024-00539-7, 38652236 PMC11133127

[76] Giannakis A Chondrogiorgi M Konitsiotis S Sidiropoulos C. Nutritional and dietary clinical trials for Parkinson's disease: a narrative review. J Neural Transmission (Vienna, Austria: 1996). (2025) 132:519–36. doi: 10.1007/s00702-025-02901-7, 40047855

[77] Yin W L F M Chen R Hultman CM Fang F Sandin S. Mediterranean diet and depression: a population-based cohort study. Int J Behav Nutr Phys Act. (2021) 18:153. doi: 10.1186/s12966-021-01227-3 34838037 PMC8627099

[78] Cabrera-Suárez BM Lahortiga-Ramos F Sayon-Orea C Hernández-Fleta JL González-Pinto A Molero P . Effect of a dietary intervention based on the mediterranean diet on the quality of life of patients recovered from depression: analysis of the predidep randomized trial. Exp Gerontol. (2023) 175:112149. doi: 10.1016/j.exger.2023.112149, 36933773

[79] Carcelén-Fraile MDC Déniz-Ramírez NDP Sabina-Campos J Aibar-Almazán A Rivas-Campo Y González-Martín AM . Exercise and nutrition in the mental health of the older adult population: a randomized controlled clinical trial. Nutrients. (2024) 16:1741. doi: 10.3390/nu16111741, 38892674 PMC11174647

[80] Ottavi TP Pepper E Bateman G Fiorentino M Brodtmann A. Consensus statement for the management of incidentally found brain white matter hyperintensities in general medical practice. Med J Aust. (2023) 219:278–84. doi: 10.5694/mja2.52079, 37604652

[81] Al-Aubaidy HA Dayan A Deseo MA Itsiopoulos C Jamil D Hadi NR . Twelve-week Mediterranean diet intervention increases citrus bioflavonoid levels and reduces inflammation in people with type 2 diabetes mellitus. Nutrients. (2021) 13:1133. doi: 10.3390/nu13041133, 33808180 PMC8065815

[82] Wang Q Wang C Abdullah Tian W Qiu Z Song M . Hydroxytyrosol alleviates dextran sulfate sodium-induced colitis by modulating inflammatory responses, intestinal barrier, and microbiome. J Agric Food Chem. (2022) 70:2241–52. doi: 10.1021/acs.jafc.1c07568 35133830

[83] Lu X Wu L Shao L Fan Y Pei Y Lu X . Adherence to the eat-lancet diet and incident depression and anxiety. Nat Commun. (2024) 15:5599. doi: 10.1038/s41467-024-49653-8, 38961069 PMC11222463

[84] Cavestro C. Metabolic dysfunction and dietary interventions in migraine management: the role of insulin resistance and neuroinflammation-a narrative and scoping review. Brain Sci. (2025) 15:474. doi: 10.3390/brainsci15050474, 40426647 PMC12109628

[85] Chen Z Zhu Y Lu M Yu L Tan S Ren T. Effects of *rosa roxburghii* tratt glycosides and quercetin on d-galactose-induced aging mice model. J Food Biochem. (2022) 46:e14425. doi: 10.1111/jfbc.14425, 36125966

[86] Zhu W Gong A Zhang B Cheng H Huang L Wu X . The chronobiological and neuroprotective mechanisms of resveratrol in improving sleep. Mediat Inflamm. (2025) 2025:4954030. doi: 10.1155/mi/4954030, 40144750 PMC11944795

[87] Lian B Zhang J Yin X Wang J Li L Ju Q . Sirt1 improves lactate homeostasis in the brain to alleviate parkinsonism via deacetylation and inhibition of pkm2. Cell Reports Med. (2024) 5:101684. doi: 10.1016/j.xcrm.2024.101684, 39128469 PMC11384727

[88] Scuto M Majzúnová M Torcitto G Antonuzzo S Rampulla F Di Fatta E . Functional food nutrients, redox resilience signaling and neurosteroids for brain health. Int J Mol Sci. (2024) 25:12155. doi: 10.3390/ijms252212155, 39596221 PMC11594618

[89] Kabra A Garg R Brimson J Ivkovi J Almawash S Ayaz M . Mechanistic insights into the role of plant polyphenols and their nano-formulations in the management of depression. Front Pharmacol. (2022) 13:1046599. doi: 10.3389/fphar.2022.1046599 36419621 PMC9676275

[90] Chen P Guo Z Lei J Wang Y. Pomegranate polyphenol punicalin ameliorates lipopolysaccharide-induced memory impairment, behavioral disorders, oxidative stress, and neuroinflammation via inhibition of tlr4-nf-кb pathway. Phytotherapy Res: Ptr. (2024) 38:3489–508. doi: 10.1002/ptr.8219, 38695373

[91] Matthews CEP Fussner LA Yaeger M Aloor JJ Reece SW Kilburg-Basnyat BJ . The prohibitin complex regulates macrophage fatty acid composition, plasma membrane packing, and lipid raft-mediated inflammatory signaling. Prostaglandins Leukot Essent Fat Acids. (2023) 190:102540. doi: 10.1016/j.plefa.2023.102540, 36706677 PMC9992117

[92] Millman JF Okamoto S Teruya T Uema T Ikematsu S Shimabukuro M . Extra-virgin olive oil and the gut-brain axis: influence on gut microbiota, mucosal immunity, and cardiometabolic and cognitive health. Nutr Rev. (2021) 79:1362–74. doi: 10.1093/nutrit/nuaa148, 33576418 PMC8581649

[93] Merlo J Chang F Tran M Alfaro J Ibrahim T Wu P . Truncated trkb: the predominant trkb isoform in nociceptors. J Pain. (2025) 31:105409. doi: 10.1016/j.jpain.2025.105409, 40280290 PMC12212927

[94] Yang Y Wang Q Wang Z Wang Y Liu B Zhang Y . The role of fatty acids in neurodegenerative diseases: mechanistic insights and therapeutic strategies. J Lipid Res. (2025) 66:100944. doi: 10.1016/j.jlr.2025.100944, 41238191 PMC12723386

[95] Serrano M Saumell-Esnaola M Ocerin G García Del Ca O G Puente N Sallés J . Impact of omega-3 on endocannabinoid system expression and function, enhancing cognition and behavior in male mice. Nutrients. (2024) 16:4344. doi: 10.3390/nu16244344 39770965 PMC11676180

[96] Kemse N Chhetri S Joshi S. Beneficial effects of dietary omega 3 polyunsaturated fatty acids on offspring brain development in gestational diabetes mellitus. Prostaglandins Leukot Essent Fat Acids. (2024) 202:102632. doi: 10.1016/j.plefa.2024.102632, 39029386

[97] Pipingas A Reddan JM Gauci S Young LM Kennedy G Rowsell R . Post-prandial cognitive and blood pressure effects of a dha-rich omega-3 powder in middle-aged males: a pilot study. Nutrients. (2023) 15:2198. doi: 10.3390/nu15092198, 37432363 PMC10181233

[98] Bej E Cesare P D'Angelo M Volpe AR Castelli V. Neuronal cell rearrangement during aging: antioxidant compounds as a potential therapeutic approach. Cells. (2024) 13:1945. doi: 10.3390/cells13231945, 39682694 PMC11639796

[99] Jara N Cifuentes M Martínez F González-Chavarría I Salazar K Ferrada L . Vitamin c deficiency reduces neurogenesis and proliferation in the svz and lateral ventricle extensions of the young guinea pig brain. Antioxidants (Basel, Switzerland). (2022) 11:2030. doi: 10.3390/antiox11102030, 36290753 PMC9598632

[100] Tschuck J Padmanabhan Nair V Galhoz A Zaratiegui C Tai H Ciceri G . Suppression of ferroptosis by vitamin a or radical-trapping antioxidants is essential for neuronal development. Nat Commun. (2024) 15:7611. doi: 10.1038/s41467-024-51996-1, 39218970 PMC11366759

[101] Menéndez SG SG Manucha W. Vitamin d as a modulator of neuroinflammation: implications for brain health. Curr Pharm Des. (2024) 30:323–32. doi: 10.2174/0113816128281314231219113942, 38303529

[102] Philippou E Hirsch MA Heyn PC van Wegen EEH Darwish H. Vitamin d and brain health in alzheimer and parkinson disease. Arch Phys Med Rehabil. (2024) 105:809–12. doi: 10.1016/j.apmr.2023.10.023, 38189701

[103] Grimm PR Tatomir A Rosenbaek LL Kim BY Li D Delpire EJ . Dietary potassium stimulates ppp1ca-ppp1r1a dephosphorylation of kidney nacl cotransporter and reduces blood pressure. J Clin Invest. (2023) 133:e158498. doi: 10.1172/JCI158498, 37676724 PMC10617769

[104] Zhang Z Yuan S Larsson SC Wang M Liu X. Serum calcium, 25-hydroxyvitamin d, and parathyroid hormone levels in relation to aneurysmal subarachnoid hemorrhage. Mol Neurobiol. (2023) 60:3004–9. doi: 10.1007/s12035-023-03254-6, 36764983

[105] Fielding RA Lustgarten MS. Impact of a whole-food, high-soluble fiber diet on the gut-muscle axis in aged mice. Nutrients. (2024) 16:1323. doi: 10.3390/nu16091323, 38732569 PMC11085703

[106] Zhou X Ma L Dong L Li D Chen F Hu X. Bamboo shoot dietary fiber alleviates gut microbiota dysbiosis and modulates liver fatty acid metabolism in mice with high-fat diet-induced obesity. Front Nutr. (2023) 10:1161698. doi: 10.3389/fnut.2023.1161698, 36969828 PMC10035599

[107] Fock E Parnova R. Mechanisms of blood-brain barrier protection by microbiota-derived short-chain fatty acids. Cells. (2023) 12:657. doi: 10.3390/cells12040657, 36831324 PMC9954192

[108] Zhao Z Geng W Gao Y Liu Y Nie S Yin Q. Effects of intermittent fasting on brain health via the gut-brain axis. Front Nutr. (2025) 12:1696733. doi: 10.3389/fnut.2025.1696733, 41356819 PMC12679884

[109] Mayneris-Perxachs J Castells-Nobau A Arnoriaga-Rodríguez M Martin M de la Vega-Correa L Zapata C . Microbiota alterations in proline metabolism impact depression. Cell Metab. (2022) 34:681–701.e10. doi: 10.1016/j.cmet.2022.04.001, 35508109

[110] Park G Kadyan S Hochuli N Pollak J Wang B Salazar G . A modified mediterranean-style diet enhances brain function via specific gut-microbiome-brain mechanisms. Gut Microbes. (2024) 16:2323752. doi: 10.1080/19490976.2024.2323752, 38444392 PMC10936641

