# Supplementary figures and images for: Research progress and hotspots of the impact of Mediterranean diet on brain health from 2005 to 2025: a bibliometric and visualization analysis
Source: Front Nutr. 2026 Mar 17;13:1796774. doi: 10.3389/fnut.2026.1796774 (PMC13035776; doi:10.3389/fnut.2026.1796774)

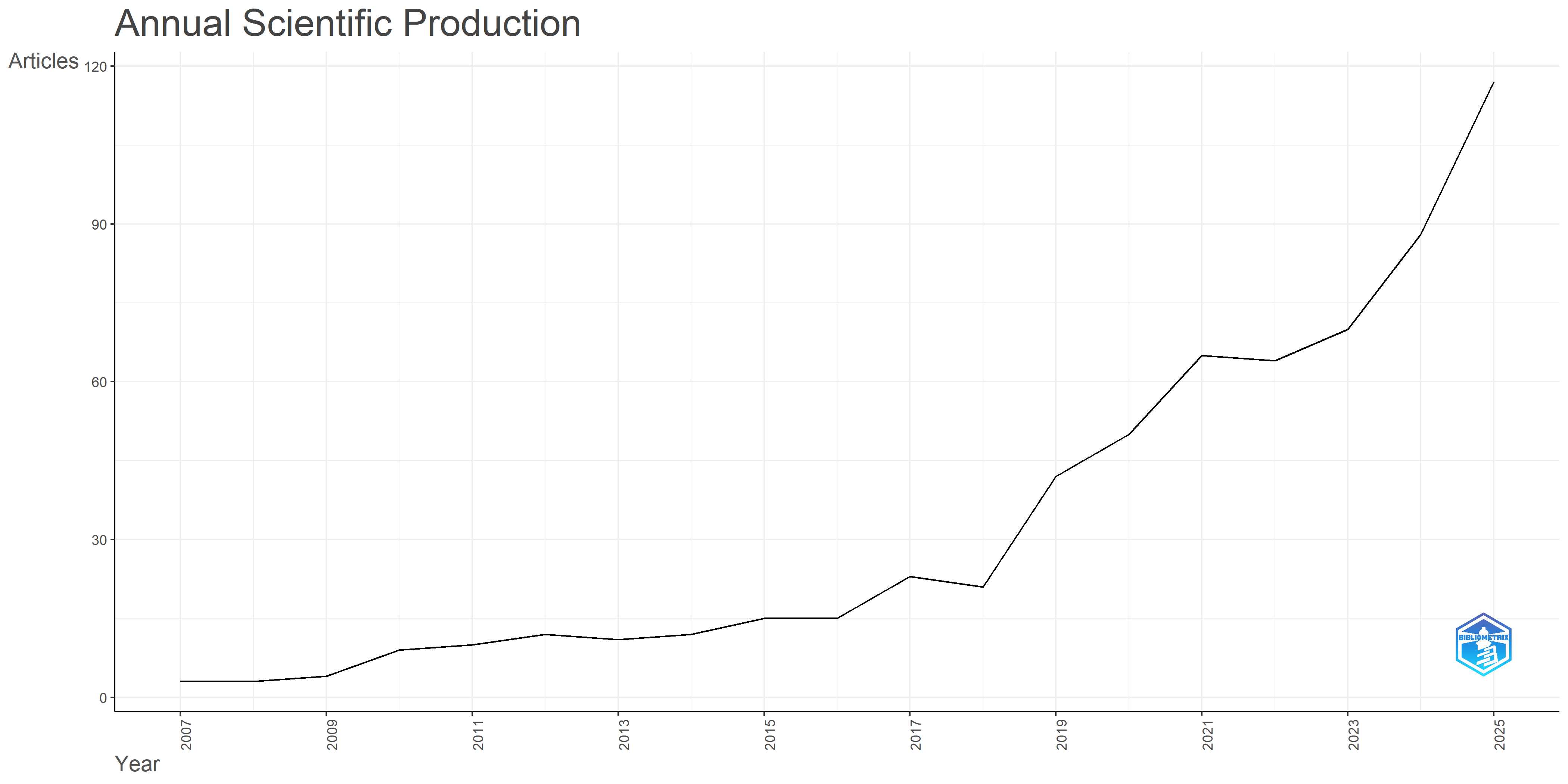

Supplement: Supplementary file 1 [file Image_1.png]

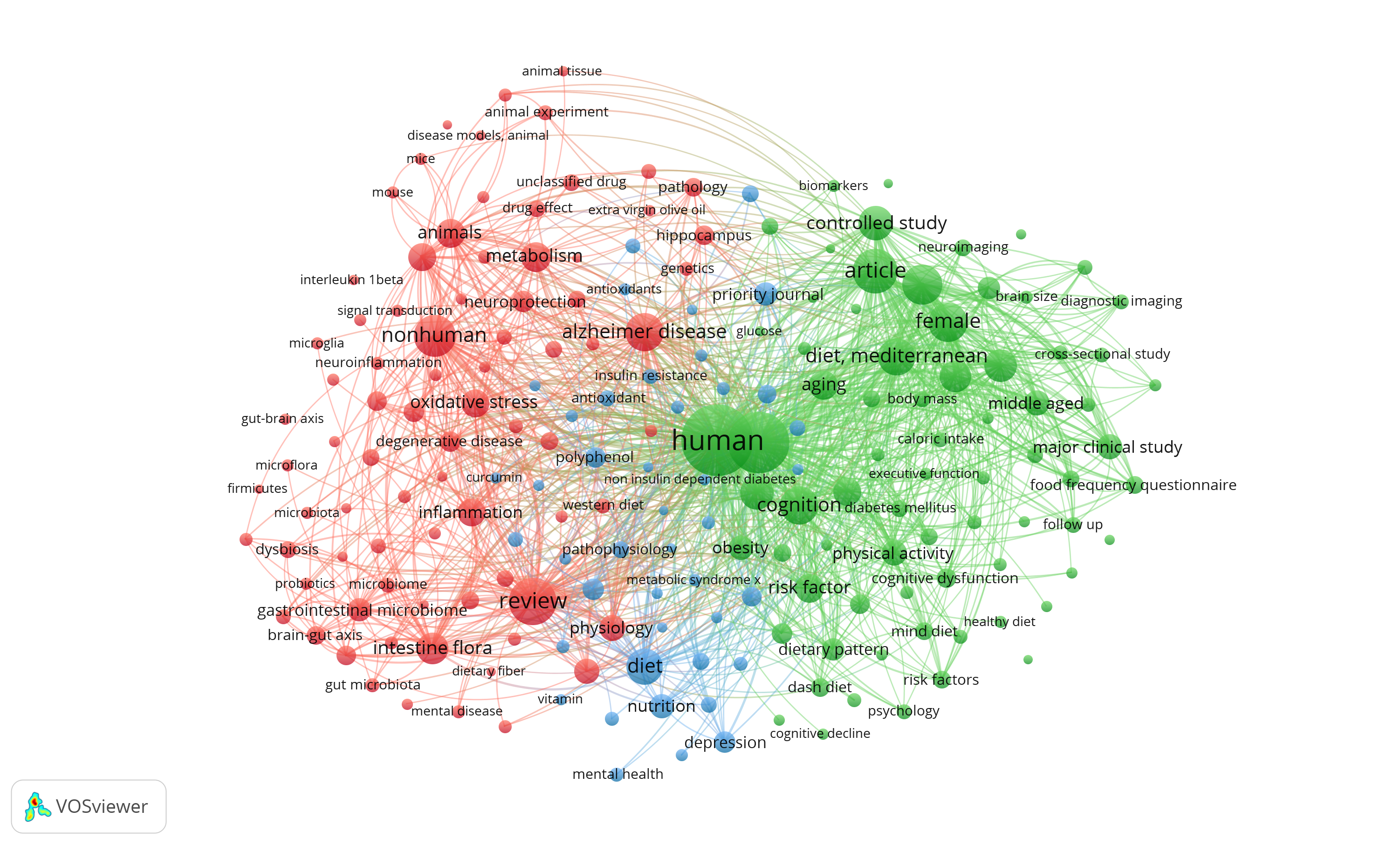

Supplement: Supplementary file 2 [file Image_2.tiff]
